# Cerium Nanoparticle‐Mediated Inhibition of the NSUN2/m^5^C Axis Suppresses Synovial Aggression in Rheumatoid Arthritis

**DOI:** 10.1002/advs.76401

**Published:** 2026-07-03

**Authors:** Ruiru Li, Simin Chen, Huijuan Hu, Shiyao Wu, Jingnan Wang, Kai Sun, Chenxi Peng, Suling Liu, Shuoyang Zhang, Yu Kuang, Wei Chen, Qian Qiu, Liuqin Liang, Yu Wen, Hanshi Xu, Youjun Xiao

**Affiliations:** ^1^ Department of Nephrology, the First Affiliated Hospital Sun Yat‐sen University Guangzhou Guangdong P. R. China; ^2^ NHC Key Laboratory of Clinical Nephrology (Sun Yat‐sen University) and Guangdong Provincial Key Laboratory of Nephrology Guangzhou Guangdong P. R. China; ^3^ Department of Rheumatology and Immunology, the First Affiliated Hospital Sun Yat‐sen University Guangzhou Guangdong P. R. China; ^4^ Department of Rheumatology and Clinical Immunology, Zhujiang Hospital Southern Medical University Guangzhou Guangdong P. R. China; ^5^ Department of Rheumatology and Immunology Department of Dermatology and Immunology National Clinical Research Center for Geriatric Disorders, Xiangya Hospital Central South University Changsha Hunan P. R. China; ^6^ Xiangya Hospital Central South University Changsha Hunan P. R. China; ^7^ Furong Laboratory Central South University Changsha Hunan P. R. China

**Keywords:** fibroblast‐like synoviocytes, ICMT, m^5^C modification, nanoparticle, NSUN2, rheumatoid arthritis, SAA

## Abstract

Rheumatoid arthritis (RA) involves persistent synovial inflammation and progressive destruction of cartilage and bone, where aberrantly activated fibroblast‐like synoviocytes (FLSs) play a central role. Although 5‐methylcytosine (m^5^C) modification is implicated in many diseases, its role in RA remains unclear. Here, we observed upregulated expression of the m^5^C methyltransferase NOP2/Sun domain family member 2 (NSUN2) in both synovial tissues (STs) and FLSs from RA patients. Functionally, NSUN2 knockdown suppressed migration and invasion of RA FLSs, whereas NSUN2 overexpression exerted opposing effects. Mechanistically, NSUN2 mediated m^5^C modification of isoprenylcysteine carboxyl methyltransferase (ICMT) mRNA and enhanced its stability. Furthermore, we identified salvianolic acid A (SAA) as an NSUN2 inhibitor, which phenocopied the effects of NSUN2 knockdown on RA FLSs. Given the well‐recognized ROS‐scavenging capacity of cerium oxide nanoparticles, we constructed Ce/SAA nanoparticles (Ce/SAA NPs) via coordination self‐assembly to enable synergistic therapy for RA. Notably, Ce/SAA NPs ameliorated arthritis in collagen‐induced arthritis (CIA) mice, and intra‐articular NSUN2‐siRNA attenuated disease progression in CIA rats. These results highlight NSUN2‑mediated m^5^C modification in RA pathogenesis and suggest NSUN2 as a therapeutic target.

## Graphical abstract

A novel epitranscriptomic mechanism in rheumatoid arthritis is uncovered: NSUN2 promotes disease via m^5^C‐dependent stabilization of ICMT mRNA, fueling the migration and invasion of pathogenic RA FLS. Targeting this axis with engineered nanoparticles (Ce/SAA NPs) effectively inhibits disease progression, presenting a precise therapeutic strategy.

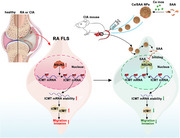

## 1 Introduction

Rheumatoid arthritis (RA) is a chronic, progressive, and destructive systemic autoimmune disease marked by persistent joint inflammation that ultimately results in irreversible joint damage [1]. The primary pathological characteristics of RA include the formation of pannus, synovitis, and the destruction of cartilage and bone. Although considerable progress has been achieved in current therapeutic strategies, a significant number of patients still show an inadequate clinical response. These unmet clinical needs highlight the necessity of elucidating the pathogenesis of RA and identifying novel therapeutic targets that can effectively inhibit synovial hyperplasia, halt cartilage degradation, and prevent bone destruction.

Fibroblast‐like synoviocytes (FLSs) derived from RA patients undergo distinctive morphological and functional transformations, acquiring “tumor‐like” phenotypic features and maintaining a persistently activated state [2], primarily due to their secretion of abundant inflammatory cytokines and matrix metalloproteinases (MMPs), along with their resistance to apoptosis, hyperproliferation, and invasive behavior. Anatomically, RA FLSs can be divided into two subsets: PDPN (podoplanin) ^+^THY1 (CD90, thymocyte differentiation antigen 1) ^−^ FLSs in the lining layer and PDPN^+^THY1^+^ FLSs in the sublining layer, representing distinct and functionally heterogeneous populations that contribute differently to synovial structure and pathology [3]. Therefore, targeting the activation of RA FLSs presents a promising therapeutic strategy, potentially reducing cartilage and bone degradation while minimizing systemic immunosuppressive side effects [4].

RNA modifications, including N6‐methyladenosine (m^6^A), 5‐methylcytosine (m^5^C), pseudouridine (Ψ), and 2’‐O‐methylation, are important post‐transcriptional modification processes. Among these modifications, m^6^A has been extensively investigated and well‐characterized, demonstrating significant roles in the pathogenesis of RA [5]. Beyond m^6^A, m^5^C is another prevalent RNA modification distributed in rRNAs, tRNAs, and mRNAs [6, 7]. The m^5^C modification is also dynamically regulated by three classes of regulators. m^5^C methyltransferases, including members of the NOP2/Sun RNA methyltransferase family (NSUN) and DNA methyltransferase (DNMT) families, catalyze m^5^C modification on RNA. Conversely, m^5^C demethylases, including the ten‐eleven translocation (TET) family, can remove such a mark. m^5^C readers, including Aly/REF export factor (ALYREF) and Y‐box binding protein 1 (YBX1), recognize m^5^C and subsequently regulate RNA processing [8]. In recent years, aberrant mRNA m^5^C modification has been reported to be involved in the pathogenesis and development of different types of cancers [6, 9, 10]. Current research has identified NSUN2 and NSUN6 as key methyltransferases that mediate maternal mRNA methylation, underscoring their potential regulatory significance in development and disease processes [11].

We report that NSUN2‐mediated m^5^C hypermethylation is elevated in RA FLSs and synovium. NSUN2 promotes RA FLSs migration by stabilizing isoprenylcysteine carboxyl methyltransferase (ICMT) mRNA in an m^5^C‐dependent manner. Using structure‐based virtual screening, we identified salvianolic acid A (SAA) as a potent and cell‐active NSUN2 inhibitor. SAA reduced m^5^C levels and inhibited RA FLSs functions in vitro.

The excessive production of reactive oxygen species (ROS) and the imbalance of the antioxidant defense system constitute the so‐called “oxidative stress,” which is widely recognized as a key driver of RA progression [12]. ROS can nonspecifically react with DNA, proteins, lipids, and extracellular matrix components, leading to dysfunction and death of synovial cells and chondrocytes, thereby directly damaging joint tissues [13]. Furthermore, ROS activates transcription factors such as NF‐κB and MAPK, promoting the expression of pro‑inflammatory cytokines including TNF‑α, IL‐1β, and IL‑6, which amplifies synovial inflammation [14, 15]. Therefore, scavenging ROS represents an effective therapeutic strategy for RA. Currently, cerium‐based nanoparticles represented by cerium dioxide (CeO_2_) have been extensively studied in various diseases associated with high oxidative stress [16, 17]. They exert an antioxidant effect by converting Ce^4^^+^ to Ce^3^^+^, thereby consuming ROS. Based on our previous experience in the preparation of metal‐coordinated nanomedicines [18, 19], overall, our findings underscore the critical role of NSUN2 and its potential as a therapeutic target for RA.

## 2 Results

### 2.1 m^5^C Hypermethylation and Upregulated NSUN2 Expression in the Synovium and FLSs From RA Patients

**FIGURE 1:**
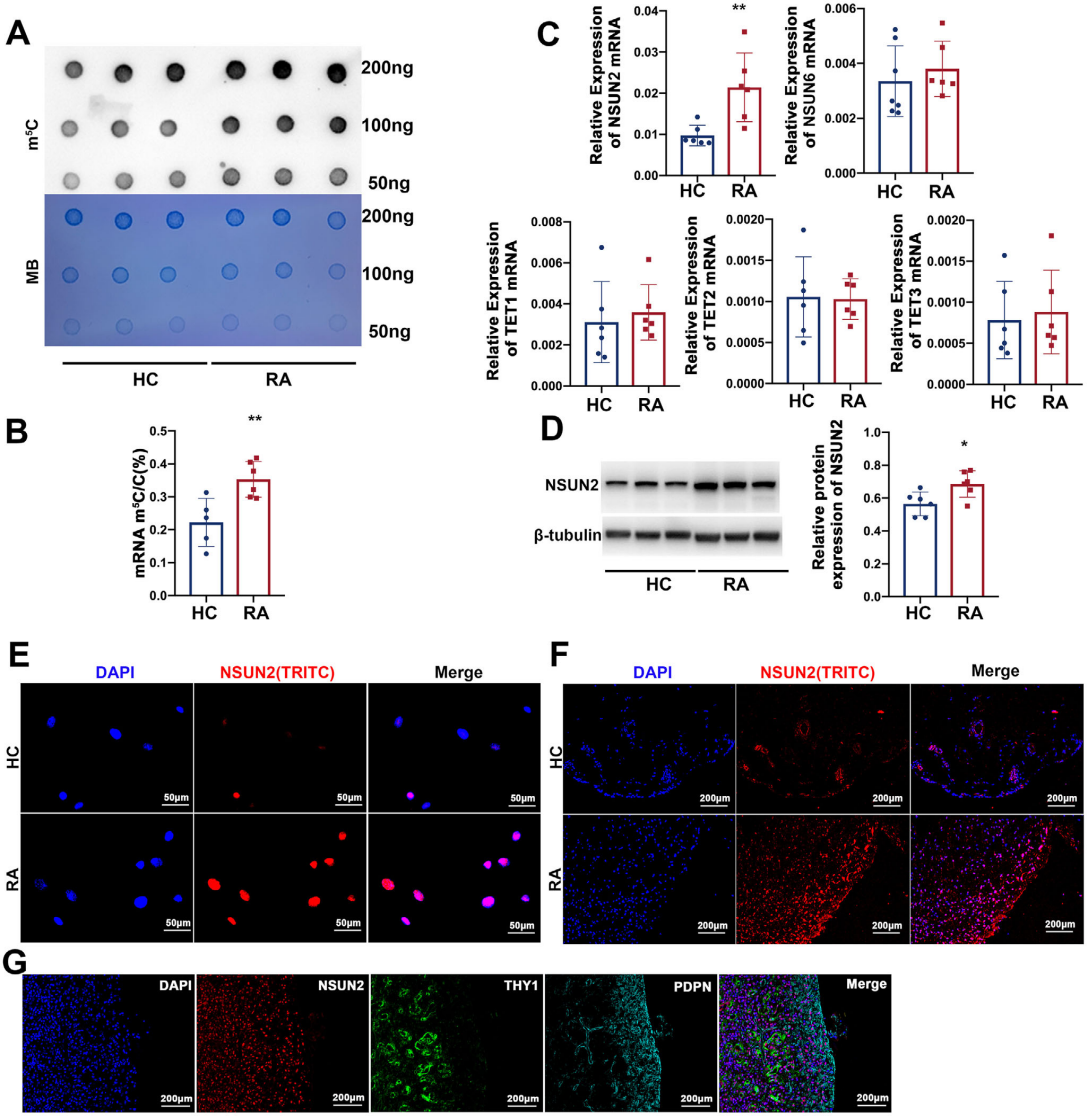
Increased NSUN2 expression in FLSs and synovial tissues from patients with RA. (A) The m^5^C level in mRNA was detected using m^5^C dot blot assays. MB, methylene blue staining (as loading control). (B) mRNA m^5^C level in RA FLSs and HC FLSs was determined by LC‐MS/MS analysis. (C) RT‐qPCR analysis of NSUN2, NSUN6, TET1, TET2, and TET3 expression in FLSs from RA patients or HC subjects. (D) Protein expression of NSUN2 in FLSs from RA patients and HC subjects was measured by Western blot analysis. The graphic indicates quantitative analysis (right panel). (E) NSUN2 expression in FLSs was detected using immunofluorescent staining (original magnification, ×400). (F) Immunofluorescence staining was used to detect NSUN2 protein expression in synovium from humans (original magnification, ×200). (G) Multiplex Immunofluorescence staining for PDPN, THY1, NSUN2, and DAPI in synovium from RA patient. PDPN and THY1 labeling highlighted the location of FLSs in synovial tissue. Data are presented as the means ± SD from at least 5 independent experiments. ^*^ *p* < 0.05, ^**^ *p* < 0.01 vs HC by Student's *t*‐test. HC: healthy control; RA: Rheumatoid arthritis, PDPN: podoplanin; THY1: CD90, thymocyte differentiation antigen 1.

We initially used m^5^C dot blot analysis to assess m^5^C levels in mRNA and found that the m^5^C content in RA FLSs was higher than that in healthy control (HC) FLSs (Figure 1A). Subsequently, liquid chromatography–tandem mass spectrometry (LC‐MS/MS) is employed to validate m^5^C levels, yielding consistent results (Figure 1B). We further examined the expression of specific methyltransferases (NSUN2, NSUN6) and demethylases (TET1, TET2, and TET3) involved in mRNA m^5^C modification. Notably, only NSUN2 exhibited significantly elevated mRNA and protein levels in RA FLSs compared to HC FLSs (Figure 1C,D). Immunofluorescence (IF) staining showed that NSUN2 was predominantly localized in the nucleus of RA FLSs (Figure 1E). To further investigate the expression patterns of NSUN2 and its clinical relevance in RA patients, we compared NSUN2 expression in synovial tissues (STs) from RA patients and HC subjects using IF staining. NSUN2 levels were significantly higher in RA synovium (Figure 1F), with prominent localization in both the lining and sublining layers (Figure 1G). Consistently, NSUN2 expression was also increased in the STs of CIA mice compared with controls (Figure S1A). We next investigated whether inflammatory factors and antirheumatic drugs regulate NSUN2 in RA FLSs. Treatment with TNF‐α or IL‐1β induced NSUN2 protein expression, whereas dexamethasone (DXM) and methotrexate (MTX) repressed it (Figure S1B). Collectively, these findings suggest that NSUN2‐mediated m^5^C methylation may contribute to RA pathogenesis.

### 2.2 NSUN2 Promotes the Migration and Invasion of RA FLSs

**FIGURE 2:**
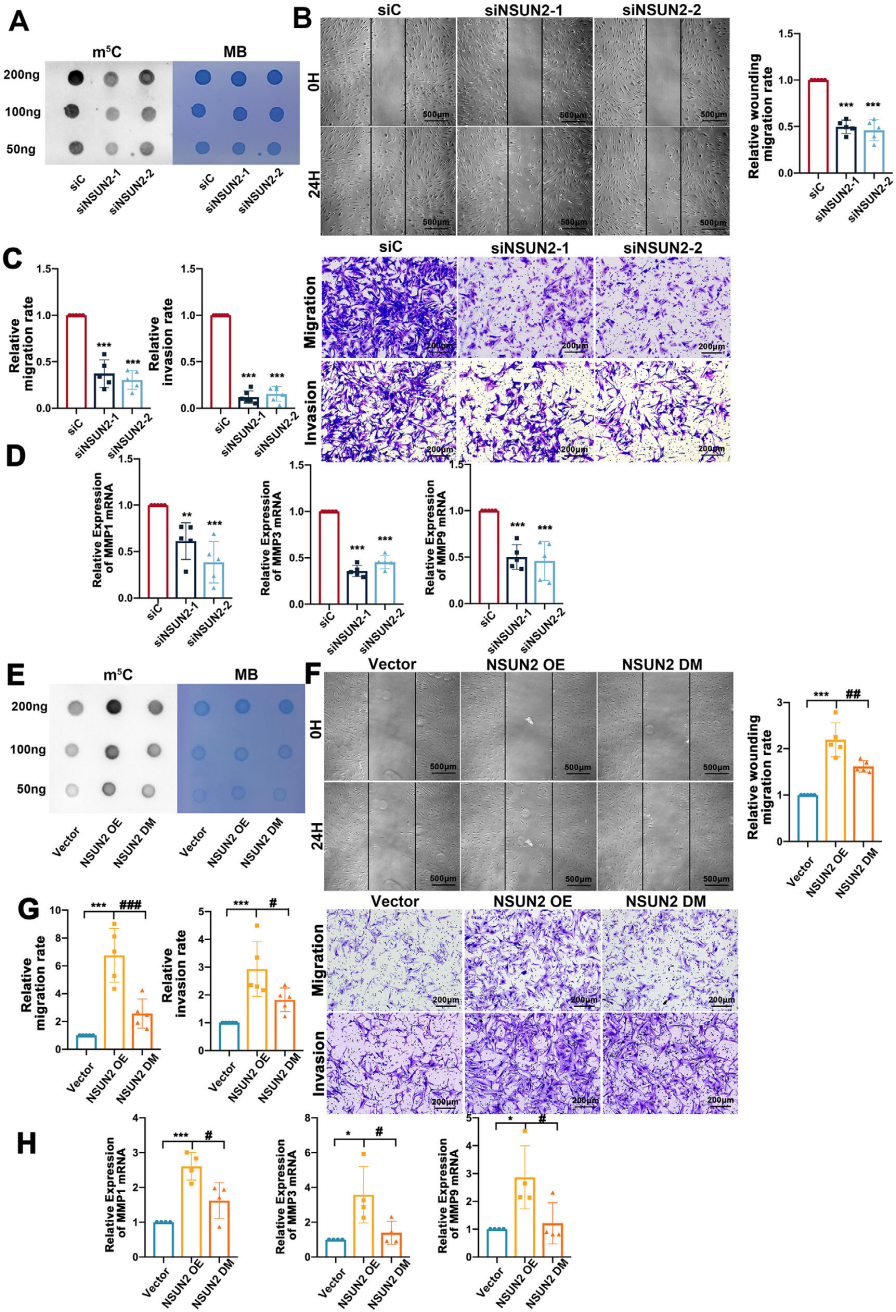
NSUN2 regulates migration and invasion of RA FLSs. (A and E) Effect of NSUN2 knockdown or overexpression on m^5^C levels, measured by dot blot assays. (B, C, F, and G) Effect of NSUN2 knockdown or overexpression on the migration and invasion of RA FLSs. Wound healing assays (B, F) showed the migratory capacity of the cells (original magnification, × 50). Transwell assays (C, G) were used to evaluate both migration and invasion (original magnification, × 100). (D and H) Effect of NSUN2 knockdown or overexpression on expression of MMPs in RA FLSs. RT‐qPCR was used to detect the expression of MMP1, MMP3, and MMP9. Data are presented as the means ± SD from at least 4 independent experiments. ^*^ *p *< 0.05, ^**^ *p *< 0.01, and ^***^ *p *< 0.001 vs. siC or Vector; ^#^ *p *< 0.05, ^##^ *p *< 0.01, ^###^ *p *< 0.001 vs. NSUN2 OE, by one‐way ANOVA with Bonferroni's posthoc comparison. MB: methylene blue staining; MMP: matrix metalloproteinase; OE: overexpression; DM: double mutant.

To investigate whether NSUN2‐mediated m^5^C hypermethylation contributes to the abnormal behaviors of RA FLSs, we first used small interfering RNA (siRNA) to silence NSUN2 expression (Figure S2A). m^5^C dot blot showed that NSUN2 knockdown reduced m^5^C levels in RA FLSs (Figure 2A). Wound‐healing assays revealed that NSUN2 knockdown impaired cell migration, showing a smaller wound closure area (Figure 2B). Consistently, transwell assays demonstrated that NSUN2 knockdown significantly suppressed both cell migration and invasion (Figure 2C).

Given the critical role of MMPs in RA FLSs invasiveness, we next examined whether NSUN2 influences MMPs expression. RT‐qPCR and ELISA showed that knockdown of NSUN2 significantly reduced the mRNA and protein secretion levels of MMP1, MMP3, and MMP9, respectively (Figure 2D and Figure S2B).

We further assessed whether NSUN2 affects proliferation, apoptosis, or inflammatory responses in RA FLSs. NSUN2 knockdown did not significantly affect cell proliferation or apoptosis rates, nor did it induce any detectable changes in the expression of inflammatory cytokines, including IL‐1β, IL‐6, and IL‐8 (Figure S2C–E).

To determine whether NSUN2 function relies on its m^5^C methyltransferase activity, we generated a catalytically inactive double mutant (NSUN2‐DM; C271A/C321A) (Figure S2F,G). Overexpression of wild‐type NSUN2 increased m^5^C levels (Figure 2E) and enhanced RA FLS migration, invasion, and MMPs expression (Figure 2F–H), whereas the NSUN2‐DM failed to elicit these effects. These findings indicate that NSUN2 regulates RA FLSs migratory capacity via m^5^C‐dependent mechanisms.

### 2.3 ICMT is the Potential Target of NSUN2

**FIGURE 3:**
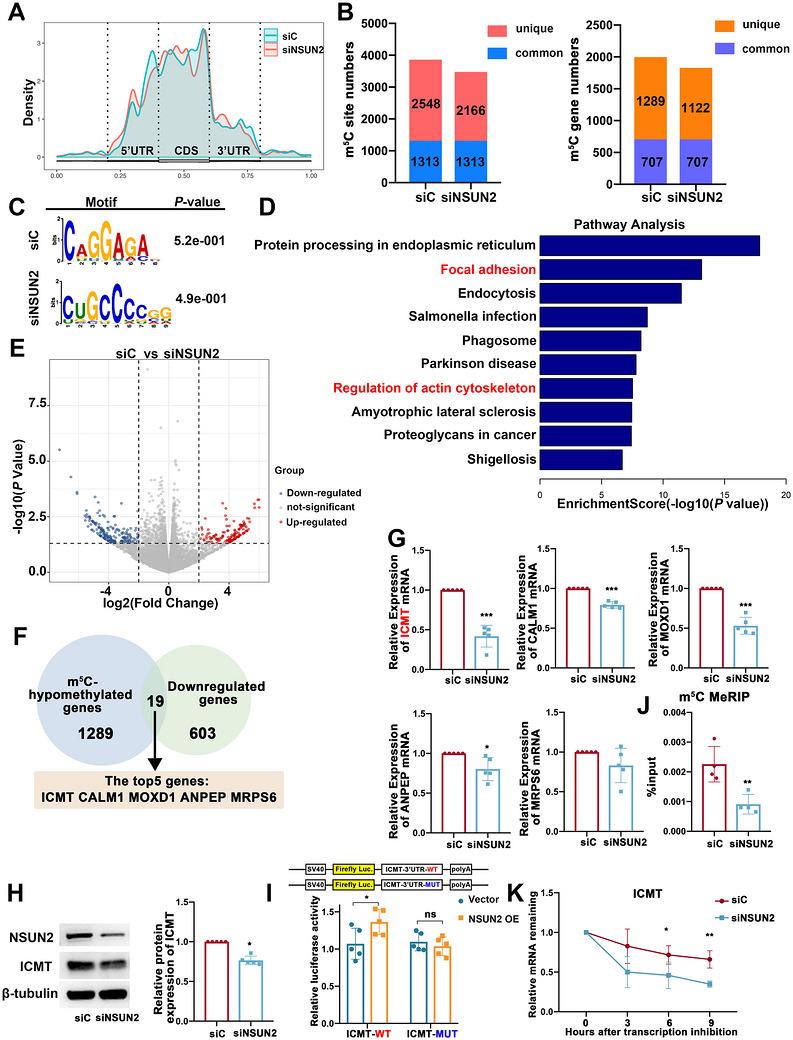
Characterization of m^5^C modification and identification of NSUN2 downstream targets in RA FLSs. (A) Distribution pattern of m^5^C sites on mRNA in RA FLSs transfected with control siRNA (siC) or NSUN2 siRNA (siNSUN2), as determined by BS‐seq. (B) BS‐seq determined the m^5^C site numbers and m^5^C genes in siC and siNSUN2 RA FLSs. (C) Top consensus motif of m^5^C sites detected by BS‐seq. (D) Bar graph showing the top 10 KEGG pathways enriched in m^5^C‐hypomethylated genes in RA FLSs. (E) Volcano plot depicting differentially expressed genes between siNSUN2 and siC RA FLSs, as determined by RNA‐Seq. (F) Venn diagram illustrating potential direct targets of NSUN2, identified by overlapping m^5^C‐hypomethylated genes with downregulated genes. (G) RT‐qPCR validation of ICMT, CALM1, MOXD1, ANPEP, and MRPS6 expression in siC and siNSUN2 RA FLSs. (H) Effect of NSUN2 knockdown on ICMT protein expression, detected by Western blot. (I) Luciferase activity of pMIR‐REPORT vectors containing wild‐type (WT) or mutant mutation at the m^5^C site ICMT 3’UTR, after infection with NSUN2 overexpression (OE) lentivirus or control vector, followed by transfection in HEK‐293T cells. (J) Relative enrichment of m^5^C modification on ICMT mRNA (normalized to input) in NSUN2‐knockdown RA FLSs, as determined by MeRIP‐qPCR. (K) Impact of NSUN2 on ICMT mRNA stability, as determined by RT‐qPCR. RA FLSs were treated with actinomycin D (5 µg/mL) and harvested at 0, 3, 6, and 9 h. Data are presented as the means ± SD from at least 4 independent experiments. ^*^ *p* < 0.05, ^**^ *p* < 0.01, and ^***^ *p* < 0.001 vs. siC or Vector+ICMT‐WT, by Student's *t*‐test. WT: wild type; MUT: mutant; OE: overexpression; ns: not significant.

To investigate how NSUN2 modulates RA FLSs functions, we performed m^5^C RNA bisulfite sequencing (BS‐seq) on mRNA isolated from FLSs of multiple RA patients. m^5^C methylation sites were located within the mRNA coding sequence (CDS) regions, 5’untranslated regions (UTR), 3’UTR of RA FLSs (Figure 3A). On average, the m^5^C methylation site numbers were significantly lower in the NSUN2 knockdown group compared to the scramble group (Figure 3B). The probability sequence context revealed a higher frequency of m^5^C sites in CG‐rich regions (Figure 3C), consistent with prior reports [20]. A total of 3861 m^5^C sites within 1996 mRNA and 3479 m^5^C sites within 1829 mRNA were identified in siC group and siNSUN2 group, respectively, with 1289 and 1122 unique genes modified in each group (Figure 3B). KEGG analysis demonstrated that 1289 m^5^C‐hypomethylated genes upon NSUN2 knockdown were significantly enriched in gene sets related to focal adhesion and the regulation of the actin cytoskeleton (Figure 3D), consistent with the role of NSUN2 in RA FLSs. We also performed RNA‐seq to assess concomitant gene expression changes (Figure 3E). Given that NSUN2‐mediated m^5^C modification promotes mRNA stability, we screened for potential target genes by overlapping m^5^C‐hypomethylated genes with downregulated genes, identifying 19 candidates. The top five downregulated hypomethylated genes were ICMT, CALM1, MOXD1, ANPEP, and MRPS6 (Figure 3F). As shown in Figure 3G, RT‐qPCR validation confirmed that NSUN2 knockdown reduced the mRNA expression of ICMT, CALM1, MOXD1, and ANPEP, but not MRPS6. Based on our m^5^C BS‐seq analysis, ICMT showed the greatest hypomethylation and was therefore selected for further validation. The concomitant reduction in ICMT protein level upon NSUN2 knockdown, as confirmed by Western blot assay (Figure 3H), collectively supports ICMT as a candidate target gene of NSUN2.

To further confirm the direct regulation of ICMT by NSUN2‐mediated m^5^C methylation, we conducted dual‐luciferase reporter assays. Luciferase activity driven by the wild‐type ICMT 3’UTR was significantly increased upon NSUN2 overexpression, whereas activity from the mutant ICMT 3’UTR remained unchanged (Figure 3I). Additionally, methylated RNA immunoprecipitation (MeRIP) combined with RT‐qPCR indicated that NSUN2 knockdown reduced m^5^C levels on ICMT mRNA (Figure 3J). Collectively, these findings demonstrate that ICMT is a direct target of NSUN2‐mediated m^5^C methylation in RA FLSs.

To test whether NSUN2 deficiency affects ICMT mRNA stability, we inhibited transcription with actinomycin D (ActD, 5 µg/mL) and monitored ICMT expression by RT‑qPCR over time. As shown in Figure 3K, NSUN2 silence reduced the stability of ICMT mRNA as evidenced by the increased mRNA degradation rate.

YBX1 and ALYREF are well‐recognized m^5^C readers and promote the stability of m^5^C‐modified mRNAs. We therefore investigated whether YBX1 and ALYREF influence ICMT mRNA expression in RA FLSs. RT‐qPCR revealed that both YBX1 and ALYREF had minimal impact on ICMT expression in RA FLSs. To further assess whether other reader‐related RBPs may be involved, we examined SRSF2, a reported mRNA m^5^C reader protein [21], LIN28B, a reported m^5^C mediator [22], and SRSF10, which was selected as a candidate RBP based on FIMO motif scanning [23]. SRSF2 and SRSF10 knockdown did not significantly alter ICMT expression, while LIN28B was barely detectable in RA FLSs. These results suggest that the tested reader‐related RBPs are not major regulators of ICMT in this context (Figure S3).

### 2.4 ICMT is Responsible for NSUN2‐Mediated Migration and Invasion of RA FLSs

**FIGURE 4:**
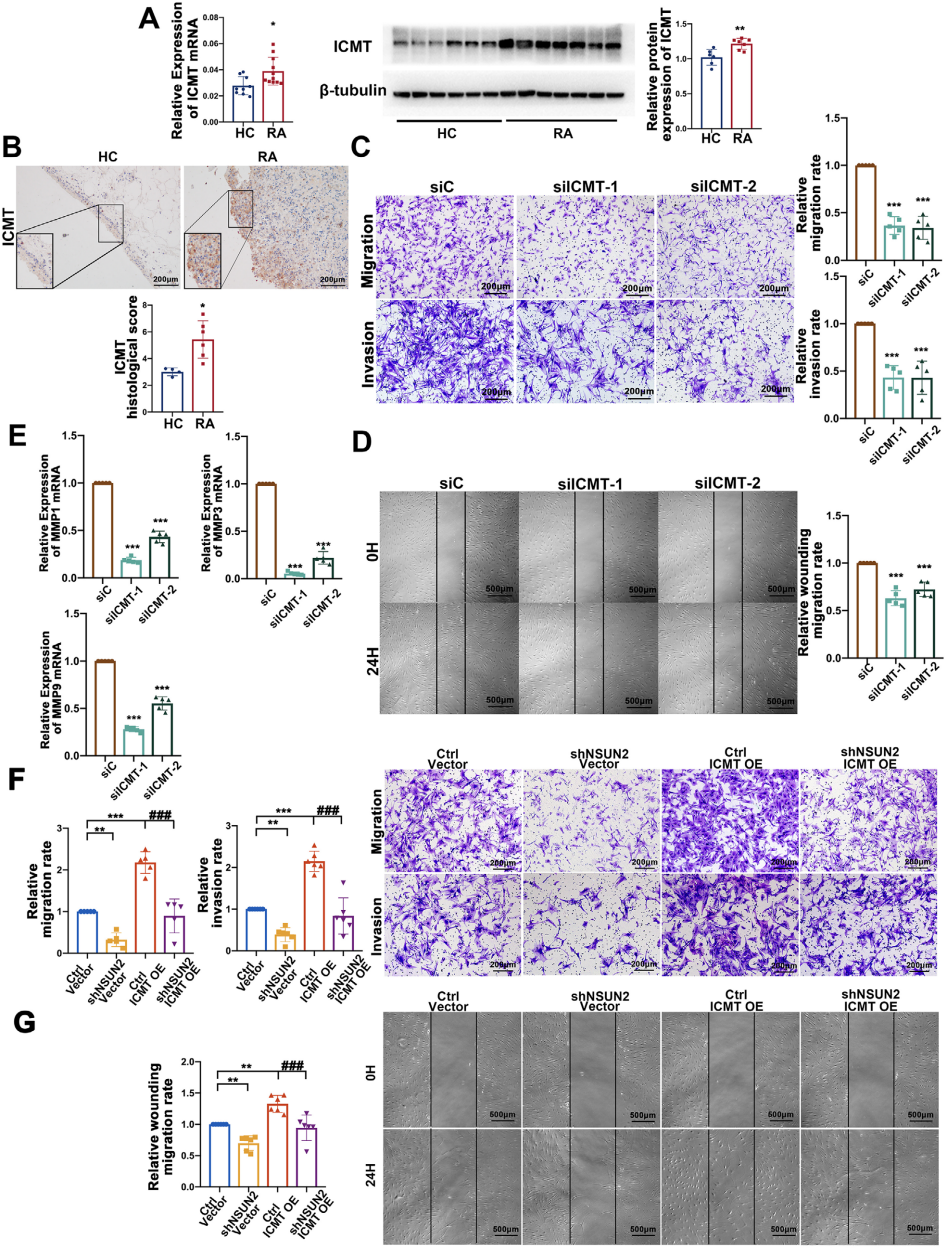
Effects of ICMT on the migration and invasion of RA FLSs. (A) ICMT mRNA and protein expression in RA FLSs and HC FLSs, detected by RT‐qPCR and Western blot (quantification shown in the right panel), respectively. (B) ICMT protein expression in human synovial tissues, analyzed by immunohistochemistry (original magnification, × 200) (quantification shown in lower panel). (C–E) Effects of ICMT knockdown on RA FLSs migration (C and D), invasion (C), and MMPs expression (E). (F–G) Effects of ICMT overexpression on NSUN2 silencing‐induced reductions in RA FLSs migration (F and G) and invasion (F) (quantification shown in left panel). Assays and treatments: Cell migration and invasion were evaluated by Transwell assays (original magnification, × 100; C and F) and wound‐healing assays (original magnification, × 50; D and G). MMP1, MMP3, and MMP9 mRNA expression was detected by RT‐qPCR. For knockdown experiments, RA FLSs were transfected with control siRNA (siC) or ICMT siRNA (siICMT) for 48 h. For rescue experiments, RA FLSs were first infected with lentiviruses encoding scramble shRNA (Ctrl), NSUN2‐targeting shRNA (shNSUN2), empty vector (Vector), or ICMT (ICMT OE) for 96 h, followed by appropriate treatments. Data are presented as the mean ± SD from at least 5 independent experiments. ^*^ *p *< 0.05, ^**^ *p *< 0.01, and ^***^ *p *< 0.001 vs. HC, siC, or Ctrl+Vector; ^###^ *p *< 0.001 vs. Ctrl+ICMT OE, by Student's *t*‐test or one‐way ANOVA with Bonferroni's post‐hoc comparison. HC: healthy control; RA: rheumatoid arthritis; MMP: matrix metalloproteinase; OE: overexpression.

To explore whether ICMT mediates the effects of NSUN2 on RA FLS function, we first examined its expression. ICMT expression was significantly upregulated at both the mRNA and protein levels in RA FLSs compared to HC FLSs (Figure 4A), and similarly elevated in RA STs vs. HC tissues (Figure 4B). Similar to the effect observed with NSUN2, treatment with TNF‐α or IL‐1β also significantly upregulated ICMT expression (Figure S4A).

Subsequently, we assessed the impact of ICMT knockdown or overexpression on RA FLSs migration, invasion, and MMPs expression. Knockdown of ICMT suppressed migration, invasion, and MMPs expression compared to control siRNA (Figure S4B–E). Conversely, ICMT overexpression enhanced these processes (Figure S4C–F). Furthermore, we conducted rescue experiments with the aim of determining whether ICMT is involved in NSUN2‐enhanced RA FLSs functions. Intriguingly, overexpression of ICMT significantly reversed the inhibitory effects of NSUN2 silencing (Figure S4G) on migration and invasion of RA FLSs (Figure 4F,G). Collectively, these data strongly indicate that ICMT mediates the NSUN2‐induced aggressive behaviours of RA FLSs.

### 2.5 SAA is a Potential Inhibitor of NSUN2 Through High‐Throughput Virtual Screening (HTVS)

To accelerate inhibitor discovery, we performed an integrated platform combining computational virtual screening and affinity‐based screening technologies. Given the absence of an experimentally resolved 3D structure for human NSUN2, we utilized a high‐confidence AlphaFold model (AF‐Q08J23‐F1), with particular focus on the domain harboring the catalytic CYS321 residue (UniProt‐reported high reliability score). Therefore, this project aims to conduct computational virtual screening of the pocket housing the active site CYS321 in the human NSUN2 protein, with the goal of identifying small‐molecule compounds that demonstrate a strong binding affinity for the target protein.

**FIGURE 5:**
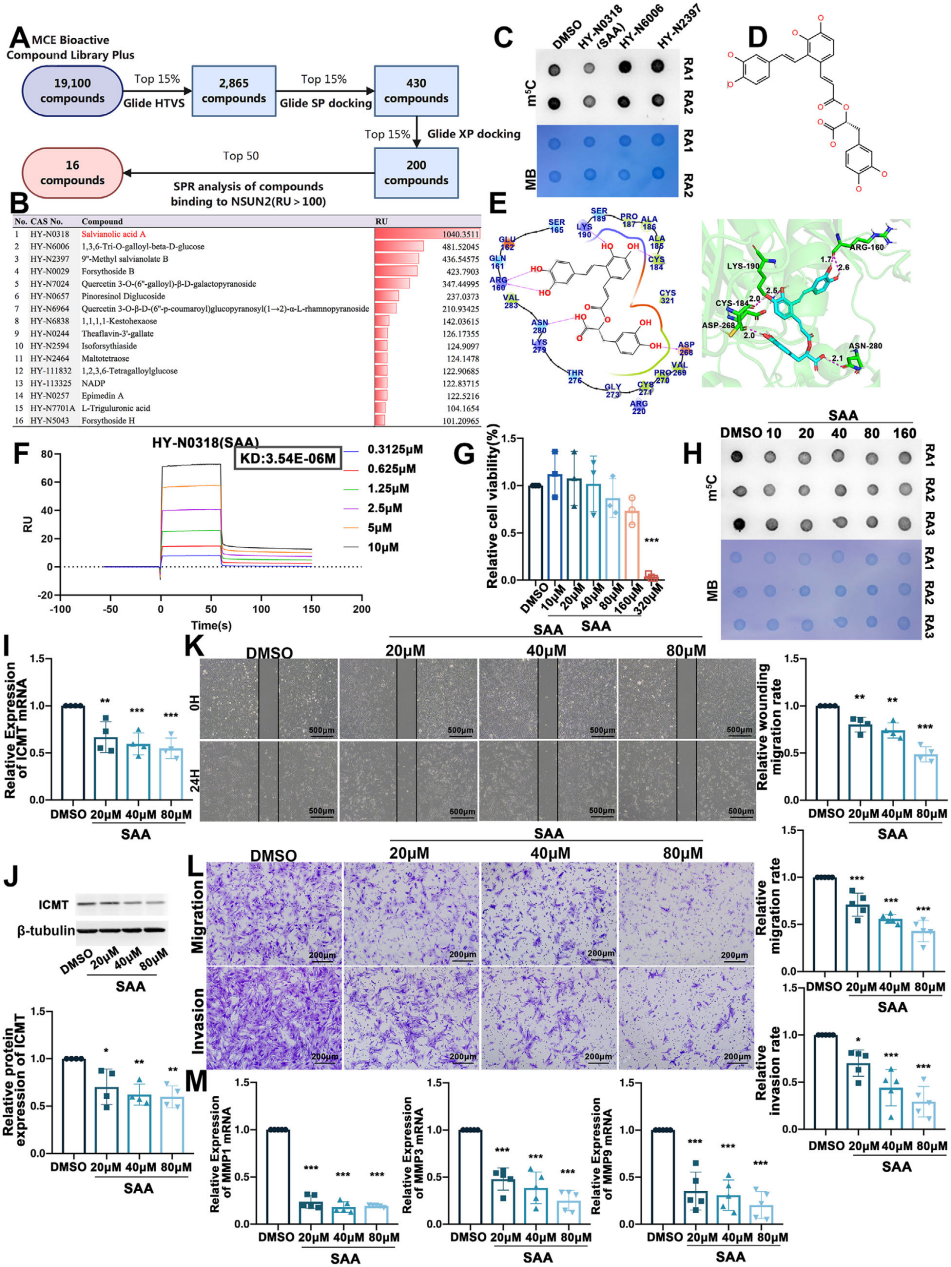
NSUN2 inhibitor Salvianolic acid A(SAA) inhibits migration and invasion of RA FLSs. (A) Schematic workflow illustrating the stepwise high‐throughput virtual screening (HTVS) process for identifying NSUN2 inhibitors. (B) Top 16 NSUN2‐binding compounds (response units [RU] > 100) selected via surface plasmon resonance (SPR) analysis from 50 docking score‐ranked candidates. (C) m^5^C methylation levels in RA FLSs treated with HY‐N0318(SAA), HY‐N6006, or HY‐N2397, detected by m^5^C dot blot assay. MB staining served as a loading control. (D) Chemical structure of SAA. (E) In silico docking model showing SAA binding to the active pocket of the human NSUN2 protein. (F) Kinetics of the NSUN2‐SAA interaction determined by SPR analysis. Curves of different colors represent binding of SAA to NSUN2 protein at varying concentrations. (G) Effect of SAA on RA FLSs viability. Cells were treated with SAA (0, 10, 20, 40, 80, 160, or 320 µM) for 24 h, and viability was assessed using the CCK‐8 assay. (H) Dose‐dependent effect of SAA (0, 10, 20, 40, 80, or 160 µM) on m^5^C levels in RA FLSs, measured by dot blot assay. (I,J) Effect of SAA on ICMT mRNA (I) and protein (J) expression in RA FLSs, analyzed by RT‐qPCR and Western blot (quantification shown in lower panel), respectively. (K,L) Dose‐dependent effects of SAA on RA FLSs migration (K and L), and invasion (L). Cells were treated with SAA (0, 20, 40, or 80 µM) for 24 h. Migration and invasion were evaluated by Transwell assays (original magnification, ×100; L) and wound‐healing assays (original magnification, × 50; K). (M) The mRNA expression levels of MMP1, MMP3, and MMP9 in RA FLSs were analyzed by RT‐qPCR (M). Data are presented as the means ± SD from at least 5 independent experiments. ^*^ *p *< 0.05, ^**^ *p *< 0.01, and ^***^ *p *< 0.001 vs. DMSO, by one‐way ANOVA with Bonferroni's posthoc comparison. HTVS: high‐throughput virtual screening; RU: resonance units; SPR: surface plasmon resonance; MMP: matrix metalloproteinase.

Following successive docking campaigns utilizing HTVS, Standard Precision (SP), and Extra Precision (XP) modes, complemented by surface plasmon resonance (SPR) assays, 16 top‐ranked compounds were selected for experimental validation (Figure 5A). Among these candidates, compounds HY‐N0318 (SAA), HY‐N6006, and HY‐N2397 exhibited the strongest binding affinity for NSUN2 (Figure 5B). To further confirm selective NSUN2 inhibitors, m^5^C dot blot assays were performed to evaluate the impact of candidate compounds on m^5^C methylation levels in RA FLSs. Among these tested compounds, only SAA significantly reduced m^5^C methylation in RA FLSs (Figure 5C). Molecular docking analysis suggested that SAA (chemical structure is shown in Figure 5D) might have a binding mode with NSUN2 protein (Figure 5E). SPR analysis further validated direct binding of SAA to NSUN2 with a dissociation constant (KD) of 3.54E‐06 M (Figure 5F). RA FLSs were treated with SAA (0–320 µM) for 24 h. Cell viability was not significantly reduced at SAA concentrations up to 160 µM, suggesting that the effects observed in subsequent experiments within this concentration range were not attributable to cytotoxicity (Figure 5G).

Dose‐response analysis further confirmed that SAA reduced m^5^C levels in a concentration‐dependent manner (Figure 5H). Additionally, SAA treatment downregulated both mRNA and protein expression of ICMT in RA FLSs in a dose‐dependent fashion (Figure 5I,J), whereas NSUN2 expression remained unaffected across all tested concentrations (Figure S5). Together, these results identify SAA as a promising candidate drug for further development of NSUN2‐targeted therapies.

We further investigated whether SAA regulates the functions of RA FLSs. As illustrated in Figure 5K,L, SAA dose‐dependently inhibited RA FLS migration and invasion, and also significantly reduced the mRNA expression of MMP1, MMP3, and MMP9 (Figure 5M).

### 2.6 Design and Characterization of the Ce/SAA NPs

**FIGURE 6:**
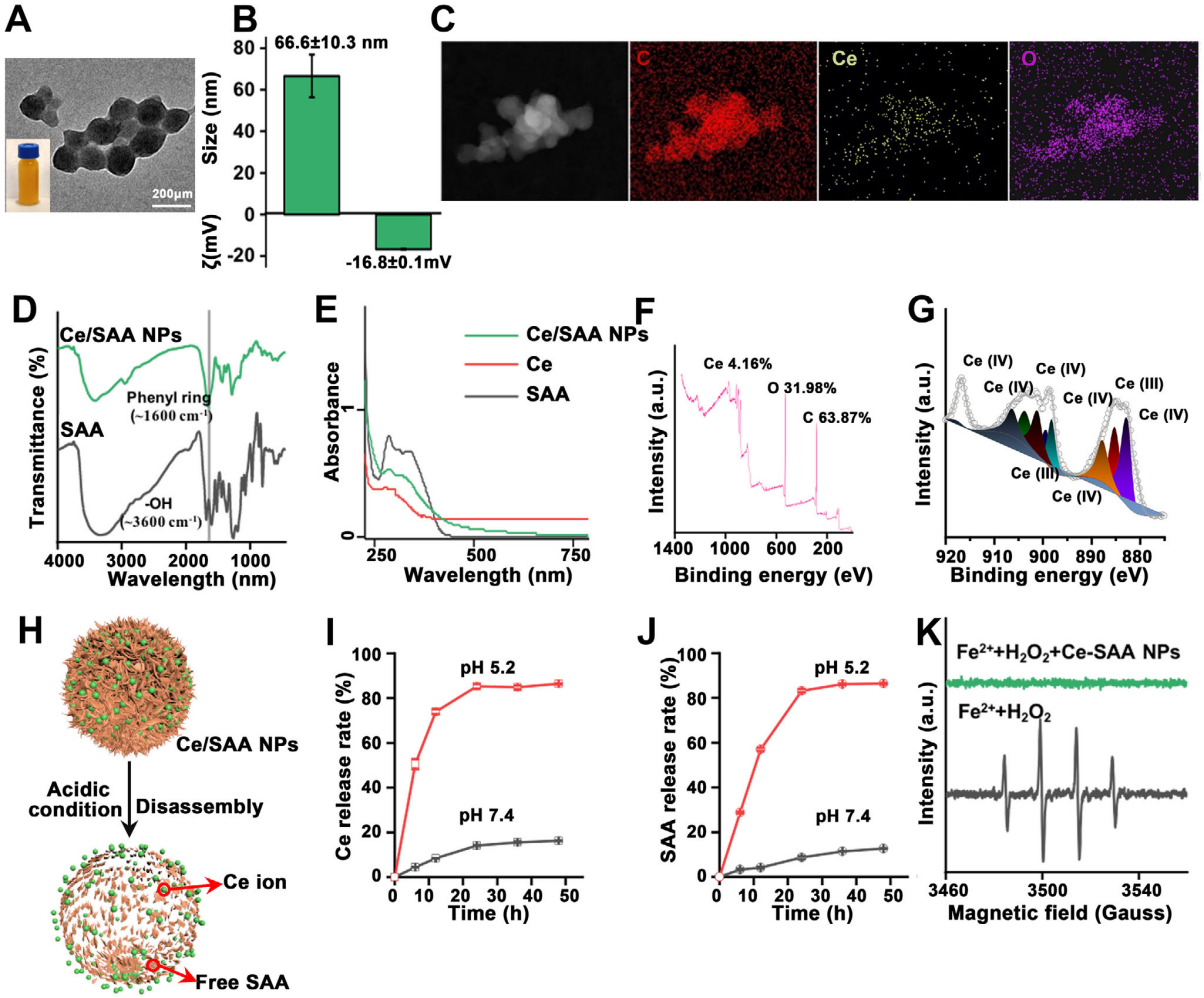
Characterization of Ce/SAA NPs. (A) TEM image of Ce/SAA NPs. (B) Hydrodynamic diameter (DLS) and zeta potential of Ce/SAA NPs in water. (C) EDS elemental mapping of Ce/SAA NPs. (D) FT‐IR spectra of SAA and Ce/SAA NPs. (E) UV–vis absorption spectra of SAA and Ce/SAA NPs. (F) XPS survey spectrum of Ce/SAA NPs. (G) High‐resolution XPS spectrum of the Ce 3d region for Ce/SAA NPs. (H) Schematic illustration of the pH‐responsive degradation of Ce/SAA NPs. (I) Cumulative Ce release from Ce/SAA NPs at different pH values. (J) Cumulative SAA release from Ce/SAA NPs under the same conditions. (K) ESR spectra of •OH generated by the Fenton reaction in the absence or presence of Ce/SAA NPs. Data are presented as the means ± SD from 3 independent experiments. SAA: salvianolic acid A; NPs: nanoparticles; TEM: transmission electron microscope; DLS: dynamic light scattering; EDS: Energy‐dispersive x‐ray spectroscopy; FT‐IR: Fourier transform infrared; XPS: X‐ray photoelectron spectroscopy; ESR: electron spin resonance.

We developed cerium‐stabilized SAA nanoparticles (Ce/SAA NPs) to enhance articular targeting. Synthesis was achieved by coordination‐driven self‐assembly of Ce^4^^+^ ions with SAA, illustrated in Figure S6A. Transmission electron microscopy (TEM) analysis revealed that the Ce/SAA NPs exhibited a uniform nanoscale spherical morphology (Figure 6A,B), with an average diameter of 66.6 ± 10.3 nm (Figure 6B,C). The nanoparticles demonstrated excellent aqueous stability with an orange color in water (Figure 6A). Energy‐dispersive x‐ray spectroscopy (EDS) mapping showed homogeneous distribution of C, O, and Ce elements throughout the nanoparticles (Figure 6C). From the Fourier transform infrared (FT‐IR) spectra in Figure 6D, phenyl ring vibration and substitution band emerge at around 1600 cm^−1^, while the peaks of ‐OH appear at 3600 cm^−1^, further validating the formation of the metallic phenolic network. UV–vis spectroscopy demonstrated a subtle blue shift in absorption peaks (288 nm → 285 nm; 340 nm → 335 nm) compared to free SAA, further verifying successful coordination (Figure 6E). X‐ray photoelectron spectroscopy (XPS) analysis confirmed the elemental composition of Ce/SAA NPs, revealing characteristic binding energies for C 1s (284.87 eV, 63.87%), O 1s (531.35 eV, 31.98%), and Ce 3d (884.08 eV, 4.16%) (Figure 6F). Deconvolution of the Ce 3d spectrum resolved two distinct oxidation states: Ce^3^^+^ (30.08%) and Ce^4^^+^ (69.92%), yielding a Ce^3^^+^: Ce^4^^+^ ratio of 3:7 (Figure 6G). The predominance of tetravalent cerium species, along with the characteristic elemental signatures, collectively confirmed the successful synthesis of Ce/SAA NPs. According to the dynamic light scattering (DLS) measurement, the zeta potential of Ce/SAA NPs suspended in water is −16.8 ± 0.1 mV, suggesting that Ce/SAA NPs are physiologically stable (Figure 6B). Furthermore, Ce/SAA NPs could be stably dispersed in PBS and DMEM over a 48 h‐incubation period, and the hydrodynamic size was nearly unchanged (Figure S6D,E), indicating that the synthesized Ce/SAA NPs have excellent stability and prolonged blood circulation. Further thermal gravity results of Ce/SAA NPs indicate that the assembled SAA accounts for 54.44% of the total weight (Figure S6F), while the loading efficiency of cerium species and SAA in Ce/SAA NPs is determined to be 20.9% and 55%, respectively, according to the elemental quantification results assayed by inductively coupled plasma mass spectrometer (ICP‐MS) or UV–vis.

Ce/SAA NPs were designed to exploit the pH‐responsive dissociation of coordination polymers, maintaining stability at physiological pH (7.4) while rapidly degrading in arthritic acidic microenvironments (pH 5.2). ICP‐MS and spectrophotometric analyses revealed pH‐dependent release kinetics: at pH 7.4, only 16.2% (SAA) and 12.6% (Ce ions) were released over 48 h, whereas at pH 5.2, release surged to 86.4% (SAA) and 86.43% (Ce ions) (Figure 6H–J). The acid‐triggered (pH‐responsive) disassembly enables targeted drug delivery to arthritic joints, thus minimizing off‐target effects and enhancing both therapeutic specificity and biosafety.

We next employed electron spin resonance (ESR) spectroscopy to further verify the •OH scavenging capability of Ce/SAA NPs. Upon addition of Fe^2^^+^ (2 mM) and H_2_O_2_ (9 mM), characteristic spectral signals of •OH radicals were observed. Significant attenuation of these spectral signals was observed following introduction of Ce/SAA NPs (50 µg/mL), confirming their efficient •OH radical scavenging performance. This antioxidant activity demonstrates Ce/SAA NPs have considerable potential for therapeutic application in RA treatment (Figure 6K).

### 2.7 In Vitro Cellular Uptake, Cytotoxicity of Ce/SAA NPs

**FIGURE 7:**
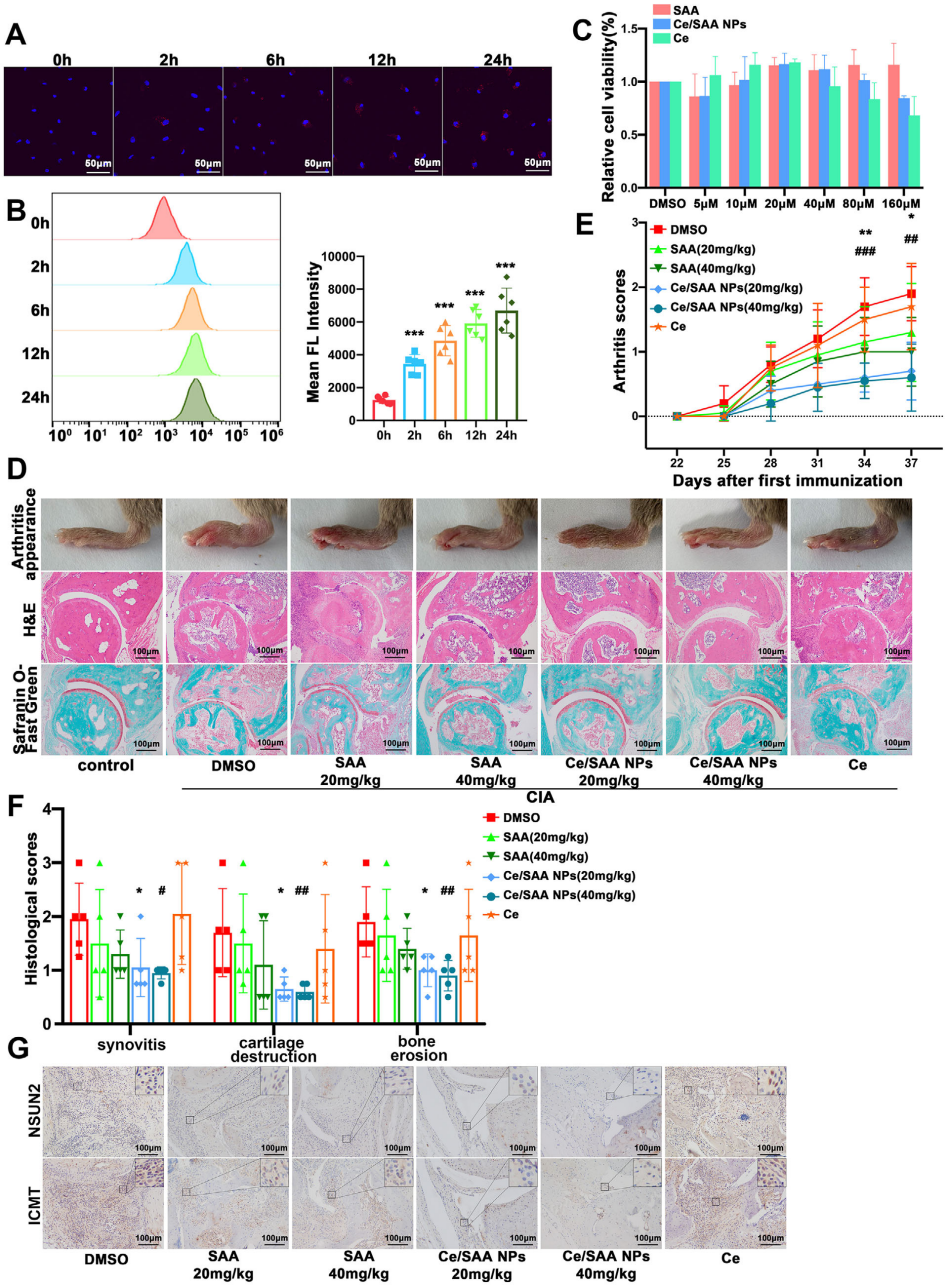
Ce/SAA NPs enhance cellular uptake and ameliorate arthritis in CIA mice. (A) Cellular uptake of Rhodamine B‐labeled Ce/SAA NPs (^RhB^Ce/SAA NPs) in RA FLSs observed by confocal laser scanning microscopy (CLSM). Red fluorescence indicates the nanoparticles (original magnification, × 400). (B) Quantitative analysis of cellular uptake efficiency measured by flow cytometry based on Rhodamine B fluorescence intensity. RA FLSs were treated with ^RhB^Ce/SAA NPs (80 µM) for 0, 2, 6, 12, and 24 h. (C) Effect of SAA, Ce/SAA NPs, and Ce on RA FLSs viability. RA FLSs were treated with SAA, Ce/SAA NPs, or Ce (0, 5, 10, 20, 40, 80, or 160 µM) for 24 h, and viability was assessed using the CCK‐8 assay. (D) Starting from day 22 postprimary immunization, CIA mice received intraperitoneal injections every other day with either DMSO, Ce alone, free SAA (20 or 40 mg/kg), or Ce/SAA NPs (20 or 40 mg/kg). Representative images showing arthritis appearance, H&E staining, and safranin O (SO)‐fast green staining of joint tissues (original magnification, × 200). (E) Quantitative analysis of clinical arthritis scores in CIA mice (*n* = 5 per group). (F) Quantitative histological scoring of joint tissues, including synovitis, cartilage destruction, and bone erosion (*n* = 5 per group). (G) NSUN2 and ICMT protein expression in STs of CIA mice, detected by immunohistochemistry (original magnification, × 200). Data are presented as the means ± SD from at least 3 independent experiments.^***^ *p *< 0.001 vs. 0 h, ^*^ *p *< 0.05, ^**^ *p *< 0.01 for group Ce/SAA NPs (20 mg/kg) vs. DMSO, and ^#^ *p *< 0.05, ^##^ *p *< 0.01, ^###^ *p *< 0.001 for group Ce/SAA NPs (40 mg/kg) vs. DMSO, by one‐way ANOVA with Bonferroni's posthoc comparison. SAA: salvianolic acid A; NPs: nanoparticles; CIA: collagen‐induced arthritis.

To investigate cellular uptake behaviours, we prepared ^RhB^Ce/SAA NPs by labeling with Rhodamine B and examined the uptake of ^RhB^Ce/SAA NPs using confocal laser scanning microscopy (CLSM). As shown in Figure 7A, red fluorescence from Rhodamine B was observed in the cytoplasm of RA FLSs after treatment with Ce/SAA NPs, with signal intensity increasing over time. The cellular uptake was quantified by flow cytometry based on Rhodamine B fluorescence intensity (Figure 7B). These results confirm that Ce/SAA NPs are efficiently internalized by RA FLSs.

We next evaluated whether nanoencapsulation influenced cytotoxicity using a CCK‐8 assay. At equivalent SAA concentrations, Ce/SAA NPs exhibited no significant difference in cytotoxicity compared to free SAA (Figure 7C), indicating that the nanoformulation does not enhance adverse effects on cell viability.

### 2.8 Ce/SAA NPs Reduce Synovial Inflammation and Bone Erosion in Mice With CIA

Following intravenous (i.v.) injection into CIA mice, our Ce/SAA NPs were primarily distributed to organs of the mononuclear phagocyte system, including the liver, spleen, and lungs, with notable accumulation also observed in the kidneys and inflamed joints (Figure S6G). This biodistribution profile is consistent with the reported behavior of various nanodelivery systems [24, 25]. Importantly, the significant enrichment of Ce/SAA NPs in the target inflamed joints corroborates previous findings on the joint‐targeting efficacy of albumin‐ceria nanoparticles [26], supporting their potential for targeted arthritis therapy.

Next, we evaluated the in vivo therapeutic efficacy of Ce/SAA NPs in CIA mice. Mice were randomly divided into seven groups (*n* = 5 per group) receiving different treatments every other day with DMSO, Ce, free SAA (20 mg/kg, 40 mg/kg), or Ce/SAA NPs (20 mg/kg, 40 mg/kg) (Figure 7D). Compared to DMSO controls, free SAA at 20 mg/kg exhibited minimal effects on CIA progression. At 40 mg/kg, free SAA exhibited a tendency toward reduced arthritis scores and amelioration of pathological features, though this reduction did not achieve statistical significance. In contrast, both dosage levels of Ce/SAA NPs (20 and 40 mg/kg) elicited markedly stronger suppression of arthritis symptoms and joint damage compared to Ce alone, highlighting the enhanced therapeutic potential of the nanoparticulate formulation (Figure 7D–F). Importantly, immunohistochemical (IHC) analysis revealed significant downregulation of NSUN2 and ICMT expression in STs following treatment with either Ce/SAA NPs (20 mg/kg and 40 mg/kg) or free SAA (40 mg/kg) (Figure 7G).

Safety assessment, including serum liver and kidney function analyses and H&E staining of liver and kidney tissues harvested on day 37, revealed no significant differences in serum biomarkers or histopathology between the SAA‐treated, Ce/SAA NP‐treated, and control groups (Figure S7), indicating favorable biocompatibility of both formulations.

### 2.9 NSUN2 Knockdown Attenuates the Severity of Arthritis in Rats With CIA

**FIGURE 8:**
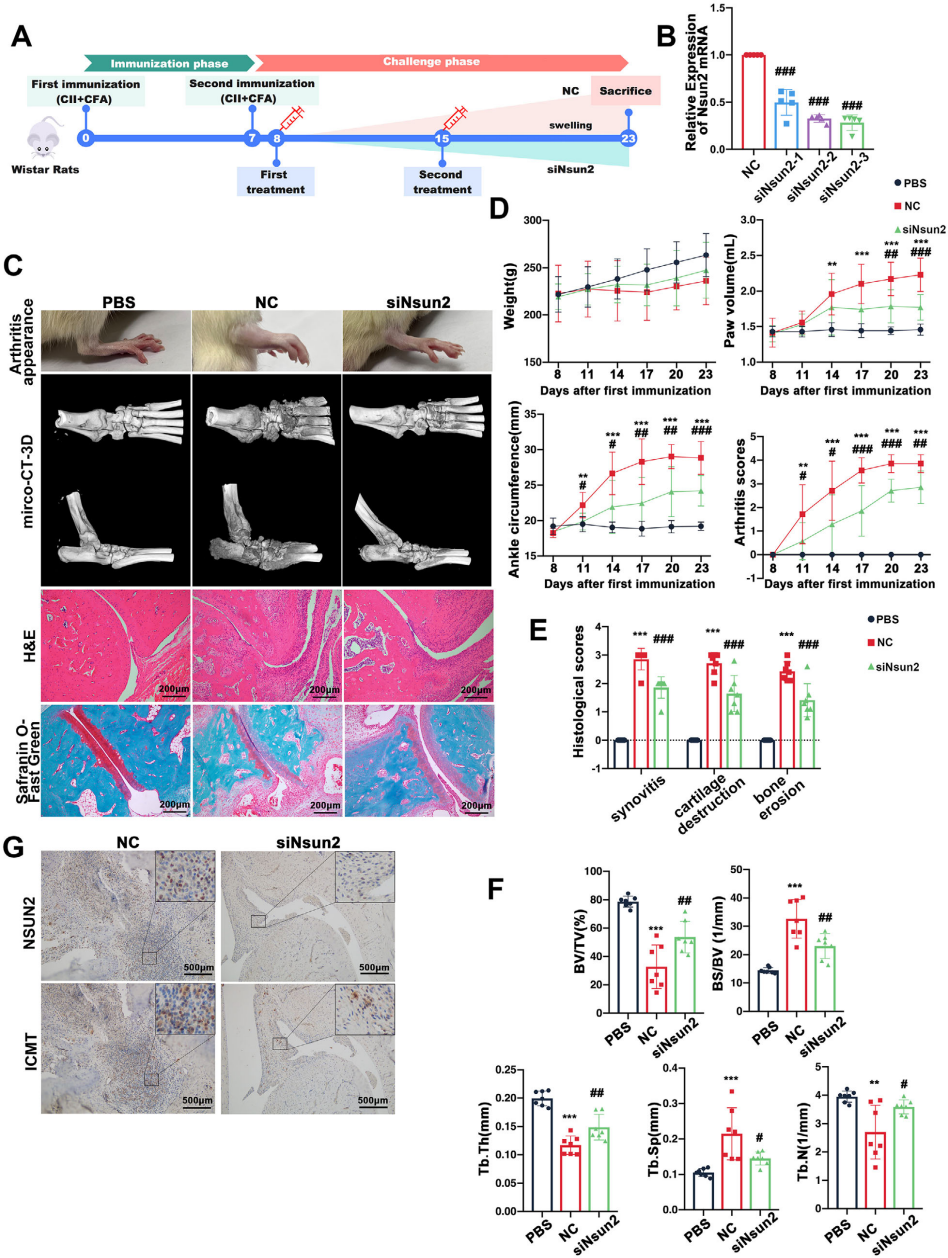
Effect of intra‐articular injection with Nsun2 siRNA on the severity of arthritis in rats with CIA. (A) Schematic illustration of the experimental design for evaluating the therapeutic effect of intra‐articular Nsun2 knockdown in a CIA rat model. (B) Knockdown efficiency of Nsun2 siRNA in CIA rat FLSs. Cells were transfected with Nsun2 siRNA for 48 h, and silencing efficiency was determined by RT‐qPCR. (C) Representative images showing arthritis phenotypes, micro‐CT scans and 3D reconstructions, H&E staining, and safranin O (SO)‐fast green staining of joint tissues (original magnification, ×100). (D) Quantitative analysis of body weight, ankle circumference, paw volume, and clinical arthritis scores in CIA rats (*n* = 7 per group). (E) Quantitative histological scoring of joint tissues, including extra‐articular infiltration, synovitis, cartilage destruction, and bone erosion (*n* = 7 per group). (F) Quantitative analysis of micro‐CT structural parameters in CIA rats (*n* = 7 per group). (G) NSUN2 and ICMT protein expression in synovial tissues of CIA rats, detected by immunohistochemistry (original magnification, ×100). (H) Proposed model for Ce/SAA nanoparticle‐mediated amelioration of rheumatoid arthritis via targeting the NSUN2‐m^5^C axis to suppress pathogenic behaviors of FLSs. Data represent means ± SD. ^**^ *p *< 0.01, ^***^ *p *< 0.001 vs. PBS, and ^#^ *p *< 0.05, ^##^ *p *< 0.01, ^###^ *p *< 0.001 vs. NC, by one‐way ANOVA with Bonferroni's post‐hoc comparison. BS: bone surface; BV: bone volume; TV: total volume; Tb.Th: Trabecular Thickness; Tb.Sp: Trabecular Separation/Spacing; Tb.N: Trabecular Number; H&E: hematoxylin‐eosin staining; CII: bovine type II collagen; CFA: complete Freund's adjuvant; SAA: salvianolic acid A; NPs: nanoparticles; CIA: collagen‐induced arthritis.

We additionally employed intra‐articular NSUN2 knockdown to clarify its functional impact on rats with CIA (Figure 8A). After designing and screening three NSUN2‐targeting siRNAs, we selected siNsun2‐3 (Figure 8B) for in vivo studies. Compared to control siRNA‐treated rats, the siNsun2‐treated group exhibited significantly reduced ankle swelling alongside marked decreases in hindlimb arthritis severity, ankle circumference, and paw volume scores (Figure 8C,D). Histological analysis demonstrated attenuated synovitis, cartilage destruction, and bone erosion (Figure 8C,E). Imaging assessments revealed substantial improvement in joint destruction (Figure 8C,F). Furthermore, IHC staining showed reduced NSUN2 and ICMT protein expression in STs of siNsun2‐treated rats (Figure 8G).

## 3 Discussion

Our data revealed the elevated levels of NSUN2 in FLSs and STs from RA patients. Functionally, NSUN2 knockdown significantly inhibited the migratory and invasive capacities of RA FLSs. Mechanistically, NSUN2 stabilizes ICMT mRNA in an m^5^C‐dependent manner to drive pathogenic functions. Through targeted screening, we identified SAA as a specific NSUN2 inhibitor. Interestingly, both intra‐articular NSUN2 silencing and Ce/SAA NPs‐mediated pharmacological inhibition attenuated synovial inflammation and bone destruction in rats or mice with CIA. Collectively, synovial NSUN2 upregulation promotes RA pathogenesis by mediating m^5^C‐dependent stabilization of ICMT mRNA.

RNA modifications have emerged as a pivotal frontier in the epigenetic field. Among them, mRNA m^5^C modification is a crucial regulator of mRNA maturation, stability, and translation, governing a wide array of physiological and pathological processes, including stress responses, embryonic development, tumorigenesis, and viral replication. For instance, NSUN2 depletion enhances type I interferon signalling during viral infections in vitro [27]. NSUN2 also promotes m^5^C modification of nuclear factor erythroid 2‑related factor 2 (NRF2), thereby augmenting NRF2 expression and antioxidative stress responses to mitigate doxorubicin‐induced myocardial injury [28]. Intriguingly, NSUN2 also stabilizes three prime repair exonuclease 2 to suppress cytosolic dsDNA accumulation and subsequent cyclic GMP‑AMP synthase (cGAS)/ stimulator of interferon genes (STING) activation, thus fostering tumor progression and resistance to anti‐PD‐L1 immunotherapy [29]. In RA, overexpression of fat mass and obesity‐associated protein (FTO) has been found to inhibit disease progression by downregulating NSUN2, suggesting a potential role of the FTO–NSUN2 axis in RA [30]. Nevertheless, the role of the NSUN2‐mediated m^5^C modification in RA remains unclear. We demonstrate that elevated NSUN2 and m^5^C enhance the migration and invasion of RA FLSs. Notably, intra‐articular siRNA targeting NSUN2 attenuated arthritis severity, synovitis, and joint destruction in CIA rats. These findings underscore the critical contribution of NSUN2/m^5^C in driving FLS‐mediated pathology, offering novel therapeutic insights for RA.

We identified ICMT as a direct target of NSUN2, with NSUN2 stabilizing ICMT mRNA. ICMT is the only known cellular enzyme that methylates the carboxyl terminus of prenylated cysteine residues, thereby acting as the terminal enzyme in the post‐translational modification cascade of CAAX‐motif proteins, including RAS family members. This function implicates ICMT in diverse physiological and pathological processes, especially oncogenesis [31]. For instance, ICMT deletion in myeloid cells and pneumocytes from mice model of K‐RAS‐induced cancer reduced myeloproliferative phenotypes and the areas of neoplastic lesions in the lungs [32]. Conversely, ICMT deficiency caused Notch1 loss‐of‐function and markedly accelerated pancreatic ductal adenocarcinoma (PDA) progression in a neoplasia model [33]. The biological function of ICMT is context‐dependent and not exclusively proto‐oncogenic, with many aspects awaiting full characterization. Our study demonstrates that NSUN2 knockdown suppresses ICMT expression, thereby attenuating the migration and invasion of RA FLSs. However, we do not completely exclude the possibility that other unidentified proteins might mediate the function of NSUN2. Further studies are also needed to clarify how ICMT regulates the migration and invasion of RA FLSs.

Our study establishes NSUN2 as a compelling therapeutic target for RA intervention. Previous pharmacological investigations have identified azetidine acrylamides as covalent NSUN2 inhibitors through cysteine‐directed activity‐based protein profiling (ABPP) [34]. To expand the repertoire of NSUN2‐targeting therapeutics, we implemented an integrative drug discovery approach combining structure‐based virtual screening of the NSUN2 catalytic pocket and high‐throughput affinity‐based screening platforms. Our results identify SAA as a novel NSUN2 inhibitor. SPR analysis revealed high‐affinity binding of SAA to critical residues within the NSUN2 m^5^C methylation active site, suggesting a competitive inhibition mechanism. This interaction was functionally validated by m^5^C dot blot analysis, demonstrating that SAA treatment significantly reduces m^5^C levels in RA FLSs. Functional assays also demonstrated that SAA effectively suppresses the migratory and invasive capabilities of RA FLSs. Notably, SAA also significantly downregulated ICMT expression in RA FLSs.

SAA, the primary bioactive constituent of Salvia miltiorrhiza, demonstrates significant therapeutic potential through its documented anti‐diabetic effects [35], nephroprotective properties [36], and capacity to attenuate atherosclerosis [37]. Although salvianolic acid B and salvianolic acid have shown efficacy in ameliorating inflammation in CIA models [38, 39], the therapeutic role of SAA in RA remained unexplored prior to this study. Notably, SAA functions as a noncovalent inhibitor of NSUN2, providing distinct advantages over covalent agents, such as lower off‐target potential and a favorable scaffold for optimization. These findings position SAA as a promising compound for the development of targeted therapies against RA through modulation of the NSUN2/m^5^C/ICMT axis.

To optimize therapeutic outcomes while mitigating potential adverse effects, we engineered a novel metal‐drug coordination nanoplatform for SAA delivery. The rationale is based on the unique features of the RA immune microenvironment. Specifically, the inflamed joint exhibits a tumor‐mimicking enhanced permeability and retention (EPR) effect, which arises from the complex network formed through interactions between stromal cells and extracellular components [40]. This EPR effect can be exploited to achieve passive accumulation of nanomaterials in inflamed joints.

Following this principle, we developed a cerium ion‐based coordination nanomedicine, designated as Ce/SAA nanoparticles (NPs), designed to harness the EPR effect for targeted SAA delivery to the joints. Under the acidic intracellular environment within the joint cavity, the Ce/SAA NPs disassemble and release the encapsulated SAA molecules. Subsequently, the lipophilic SAA passively diffuses into the nucleus, where it inhibits NSUN2. Importantly, the nuclear delivery of SAA does not rely on any nucleus‐specific receptor; instead, it is achieved through passive diffusion of the small lipophilic molecule released from the nanoparticles in the cytoplasm.

Our experimental data revealed that SAA delivered via this nanoplatform exhibited potent therapeutic effects, and correspondingly, Ce/SAA NPs were shown to effectively attenuate joint damage in collagen‐induced arthritis (CIA) models, highlighting their potential for clinical translation. It should be noted that although SAA is a specific NSUN2 inhibitor identified through our screening, all small‐molecule inhibitors may have certain non‐specific effects, including global m^5^C methylation disturbance and potential off‐target effects. This limitation awaits further validation and improvement in subsequent experiments.

In summary, this work delineates a novel epitranscriptomic pathway involved in the pathogenesis of RA and establishes the inhibition of NSUN2 as a promising therapeutic strategy. Furthermore, the development of SAA and its nanoformulation offers a framework for translating RNA modification research into clinical applications for autoimmune diseases.

## 4 Conclusions

In summary, this study establishes that NSUN2‐mediated mRNA m^5^C methylation functions as a critical epigenetic driver of FLSs activation in RA. We delineated a complete pathogenic axis wherein NSUN2 upregulation enhances the stability of ICMT mRNA via m^5^C modification, thereby fueling the migratory and invasive properties of RA FLSs. With the aim of therapeutic application, we identified SAA as a direct NSUN2 inhibitor and further engineered acid‐responsive cerium‐based nanoparticles (Ce/SAA NPs) for targeted delivery. Genetic knockdown of NSUN2 and pharmacological inhibition by SAA effectively suppressed FLS pathogenicity in vitro; moreover, both NSUN2 knockdown and therapeutic administration of Ce/SAA NPs alleviated arthritis severity in vivo. Collectively, our findings reveal a previously unrecognized role of mRNA epitranscriptomics in synovial pathology, nominate NSUN2 as a viable therapeutic target for RA, and provide a promising nanomedicine‐based strategy for precise epigenetic intervention. This work advances the understanding of RA pathogenesis into the realm of epitranscriptomic regulation and opens new avenues for developing disease‐modifying therapies.

## 5 Materials and Methods

### 5.1 Study Design

The objectives of this study were to investigate the role of the m^5^C methyltransferase NSUN2 in the pathogenesis of RA and to elucidate the underlying molecular mechanisms. The study was organized into four main components to address these objectives. First, primary RA FLSs were utilized to assess the functional effects of NSUN2. Second, the primary targets of NSUN2 were identified through bisulfite sequencing (BS‐seq) and RNA sequencing (RNA‐seq). Third, SAA was screened and validated as an inhibitor of NSUN2. Finally, the therapeutic potential of NSUN2 inhibition was evaluated using either cerium‐SAA nanoparticles(Ce/SAA NPs) or intra‐articular injection of NSUN2‐siRNA in CIA models in mice or rats. In vitro assays included RT‐qPCR, EdU proliferation assays, Annexin V/PI apoptosis detection, and Transwell migration and invasion assays. All cellular experiments were performed using RA FLSs with knockdown or overexpression of NSUN2 and ICMT, with a minimum of three independent replicates. Exact replicate numbers are specified in the figure legends. Immunohistochemical analyses were quantified by two independent pathologists who were blinded to the experimental conditions. For animal studies, subjects were randomly allocated to treatment groups. Sample sizes (*n* = 5 or *n* = 7) per group, are provided in the figure legends. Detailed descriptions of the experimental design, methodologies, and statistical analyses are available in the figures, figure legends, and supporting materials. This study involving human participants was approved by the IEC for Clinical Research and Animal Trials of the First Affiliated Hospital of Sun Yat‐sen University (Application no. [2023]816). Informed consent was obtained from all participants prior to their involvement. Animal experiments were approved by the Animal Experimental Ethics Committee of Sun Yat‐sen University (No. SYSU‐IACUC‐2023‐001236 and No. SYSU‐IACUC‐2024‐002809).

### 5.2 STs and FLSs

STs were obtained from RA patients, comprising 21 females and 4 males with an average age of 56.88 ± 10.505 years, who underwent total knee replacement or synovectomy at the First Affiliated Hospital of Sun Yat‐sen University. All cases met the 2010 American College of Rheumatology/European League Against Rheumatism (ACR/EULAR) classification criteria. Healthy controls (HC) synovial specimens were obtained from individuals undergoing traumatic above‐knee amputation, with no history of acute or chronic arthritis.

Baseline demographics and clinical features of the RA patients and HC subjects are presented in Tables S1 and S2. No significant differences in sex or age were observed between RA patients and HC subjects. All participants provided written informed consent. The study involving human samples was approved by the ethics committees of the First Affiliated Hospital of Sun Yat‐sen University.

Samples were washed with PBS, minced under sterile conditions, and enzymatically digested with 1 mg/mL collagenase at 37°C for 3 h to isolate synoviocytes. Cells were cultured in DMEM supplemented with 10% FBS at 37°C in a 5% CO_2_ incubator and passaged using trypsin when reaching 80%–90% confluence. Synoviocytes from passages 3–6 were employed for all experiments.

### 5.3 m^5^C Dot Blot

Total RNA was isolated using TRIzol reagent (Sigma‐Aldrich, USA, T9424), and mRNA enrichment was achieved with an mRNA enrichment kit (Bio‐linkedin, China, NK‐1001) according to the manufacturer's instructions. The m^5^C dot blot assay was conducted following the previously described protocol [24]. Briefly, after denaturation at 95°C for 3 min, the RNA samples were loaded onto an Amersham Hybond N+ membrane (GE Healthcare, USA, RPN203B) and subjected to crosslinking twice under UV light using an SGLinker UV Crosslinker (SinSage, China, 700101). The membrane was then blocked with 5% nonfat milk at room temperature for 1 h and probed overnight at 4°C with an anti‐m^5^C antibody (1:500 dilution, Abcam, UK, ab10805), followed by incubation with HRP‐conjugated secondary anti‐mouse IgG (1:10000 dilution, Cell Signaling Technology, USA, 7076) after washing three times with 0.1% TBST. Dot blots were measured and photographed using a chemiluminescence imaging system.

### 5.4 Liquid Chromatography–Tandem Mass Spectrometry (LC–MS/MS)

Total m^5^C levels in purified mRNA were quantified using LC‐MS/MS (Metware Biotechnology, Wuhan, China). Briefly, mRNA was enzymatically digested into nucleosides using S1 nuclease (Takara, Japan, 2410A), alkaline phosphatase (Takara, Japan, 2250A), and phosphodiesterase I (Sigma‐Aldrich, USA, P3243) at 37°C. The digest was extracted with chloroform, centrifuged, and the aqueous phase was analyzed by UPLC‐ESI‐MS/MS. m^5^C was quantified via peak integration against an external standard curve.

### 5.5 Lentivirus Infection

We acquired lentivirus particles for NSUN2 knockdown (NSUN2‐shRNA), NSUN2 overexpression (NSUN2‐OE), NSUN2 enzyme activity mutant overexpression (NSUN2‐MUT), and ICMT overexpression (ICMT‐OE) from Genechem (Shanghai, China). Lentiviral infection was conducted following the manufacturer's protocol. In brief, when reaching 50% confluence, RA FLSs were treated with lentivirus particles at a multiplicity of infection (MOI) of 40, together with HitransG P. 16 h after infection, the supernatant was removed, and the cells were cultured for an additional 3 days prior to any further processing. The efficiency of knockdown and overexpression was confirmed using real‐time quantitative PCR (RT‐qPCR) and Western blot assays. The target sequences of the shRNAs are listed in the Table S3.

### 5.6 siRNA Transfection

NSUN2 siRNA (siNSUN2), ICMT siRNA (siICMT), YBX1 siRNA (siYBX1), ALYREF siRNA (siALYREF), Nsun2 siRNA (siNsun2), and negative control siRNA (siC) were obtained from RiboBio (Guangzhou, China) or Tsingke (Beijing, China). For transfection, RA FLSs were seeded at 60%‐70% confluence in 6‐well plates (NEST Biotechnology, Wuxi, China, 703001), and then transiently transfected with 100 nM siRNA using Lipo3000 (Thermo Fisher Scientific, USA, L3000015) following the manufacturer's protocol. The transfection efficacy was determined with RT‐qPCR and Western blot assay after the indicated time. Experiments were carried out 48 h post‐transfection. The target sequences of the siRNAs are listed in the Table S4.

### 5.7 RT‐qPCR

Total RNA was purified using TRIzol reagent (Sigma‐Aldrich, USA, T9424) according to the manufacturer's instructions. Then, the RNA was reverse‐transcribed into cDNA using PrimeScript RT Master Mix (Agbio, China, AG11706), and quantitative PCR (qPCR) was performed using SYBR Green Premix Pro Taq HS qPCR Kit (Agbio, China, AG11701) on a CFX96 Touch Real‐Time PCR Detection System (Bio‐Rad, USA, CFX96 Touch) or Roche Lightcycler480 real‐time PCR systems (Roche, USA, LightCycler 480 II), following the manufacturer's guidelines. Cycle threshold (Ct) values for each gene were quantified and normalized to the endogenous reference (ΔCt = Ct target gene‐Ct reference gene), with subsequent determination using the ΔΔCt method (ΔΔCt = ΔCt sample‐ΔCt calibrator). β‐actin served as the endogenous control. All samples were analyzed in duplicate. Primer sequences are provided in Table S5.

### 5.8 ELISA

The concentrations of MMPs in the supernatants were detected by ELISA according to the manufacturer's instructions (Bioswamp, Wuhan, China; MMP‐1: SHM10740, MMP‐3: SHM10736, MMP‐9: SHM10095).

### 5.9 Western Blot Assay

Cells were grown to confluence, harvested, and lysed with radioimmunoprecipitation assay lysis buffer (50 mM TrisHCl, pH 7.4, 150 mM NaCl, 1% NP‐40, and complete protease inhibitor cocktail). Protein concentration in the whole cell lysates was quantified using BCA protein assay (Beyotime, China, P0011). Equal amounts of protein from each sample were separated by SDS‐PAGE and transferred onto nitrocellulose membranes for immunoblotting. Then the membranes were blocked with 5% nonfat milk for 2 h at room temperature, probed with appropriate primary antibodies overnight at 4°C, and incubated with corresponding secondary antibodies for 1 h at room temperature. Finally, protein bands were visualized by using ECL reagent (Millipore, USA, WBKLS0500). The antibodies used in this study were NSUN2 (Abcam, UK, ab259941), ICMT (Immunoway, USA, YN8330), and tubulin (ABclonal, China, A12289).

### 5.10 EdU Assay

Proliferation of RA FLSs was detected by EdU (5‐ethynyl‐2’‐deoxyuridine) assay using a Cell‐Light EdU DNA Cell Proliferation Kit (Beyotime, China, C0075) according to the manufacturer's instructions. Cell reaching 80% to 90% confluence were starved overnight in serum‐free medium. After incubation with 50 µM EdU in complete media for 24 h, cultured cells were fixed with 4% paraformaldehyde for 15 min, washed three times with PBS buffer, permeabilized with 0.1% TritonX‐100/PBS, following three PBS washes, incubated with Click reaction solution for 30 min, and stained with 100 µL Hoechst 33342 (1:1000 dilution) for another 10 min. The EdU‐positive FLSs were photographed and counted under a fluorescence microscope (Leica, Germany, DMI8).

### 5.11 Transwell Assay

Cell migration and invasion were detected by transwell assays. For the migration assay, FLSs were seeded at a final concentration of 6 × 10^4^ cells/mL in serum‐free DMEM in the upper chamber of a diameter of 6.5 mm and a pore size of 8.0 µm filter. DMEM containing 20% FBS was added in the lower chamber as a chemoattractant. Following incubation for 8 h, the non‐migrating FLSs remaining on the top surface of the filter were gently scraped off with a cotton swab, while migrated FLSs were fixed with methanol for 15 min and then stained with 0.1% crystal violet for 15 min. The cell migration rate was quantified and counted as the mean number of cells per 10 random fields for each assay using bright‐field microscopy. For the in vitro invasion assay, identical procedures were performed using Matrigel‐coated inserts (BD Biosciences, USA, 356234), with the matrix pre‐applied to upper chamber filters.

### 5.12 Wound‐Healing Assay

For the wound healing assay, RA FLSs were seeded on 35‐mm culture dishes and grown until 80%–90% confluent monolayer. To create an artificial standardized wound, monolayer RA FLSs were then scratched with a 200‐µL sterile pipette tip, washed three times with PBS to remove debris, and then cultured in fresh complete DMEM‐medium to facilitate cell migration at 37 °C for 24 h. Following 24 h incubation, cell migration was quantified by counting cells that had moved beyond a reference line.

### 5.13 Cell Apoptosis Assay

Apoptotic cells were determined by Annexin V‐APC and PI staining (GOONIEBIO, China, 100–102) and analyzed by flow cytometry. Briefly, the harvested cells were gently resuspended in binding buffer, and then dual‐stained with 5 µL Annexin V and 5 µL PI for 15 min in darkness at room temperature. Afterwards, samples were analyzed by flow cytometry within 1 h of staining.

### 5.14 CCK‐8 Assay

The viability of RA FLSs was evaluated using a CCK‐8 assay. Cells were pre‐exposed to SAA at concentrations ranging from 0 to 320 µM for 24 h. Subsequently, the culture supernatants were removed and replaced with 100 µL of fresh medium containing 10% CCK‐8 reagent (DOJINDO, Japan, CK04) per well. The plates were then incubated at 37°C for 4 h, and the absorbance at 450 nm was measured using a microplate reader to determine the number of viable cells.

### 5.15 Immunofluorescence Analysis

For cellular immunofluorescence, coverslips‐grown RA FLSs were fixed with 4% paraformaldehyde for 15 min, permeabilized with 0.1% Triton X‐100 in PBS for 15 min, then blocked with 5% BSA for 30 min and subsequently incubated with primary antibodies against NSUN2 (Abcam, UK, ab259941), followed by Cy3‐conjugated secondary antibody (Abclonal, China, AS007) at room temperature for 1 h. Next, cell nuclei were stained with dye 4’6‐diamidino‐2‐phenylindole dihydrochloride (DAPI) at room temperature for 10 min.

For tissue immunofluorescence staining, histological sections were deparaffinized, rehydrated, and subjected to heat‐mediated antigen retrieval in citric acid buffer. The sections were blocked with 5% BSA in PBS for 1 h, then incubated overnight at 4°C with primary antibodies against NSUN2 (Abcam, UK, ab259941), PDPN (Proteintech, China, 11629‐1‐AP), and THY1 (Proteintech, China, 66766‐1‐Ig). After primary antibody incubation, sections were incubated with an HRP‐conjugated secondary antibody, followed by signal amplification using the Tyramide Signal Amplification (TSA) plus Fluorescein System (Servicebio, China, G1236) according to the manufacturer's instructions. Finally, cell nuclei were stained with DAPI, and images were acquired using a microscope (Olympus, Japan, BX63).

### 5.16 RNA BS‐seq

In brief, total RNA was extracted from RA FLSs using the Trizol method (ThermoFisher, USA, Cat. No. 15596026CN). RNA concentration and integrity (RIN) were assessed with a BioAnalyzer 2100 system (Agilent Technologies, USA). Ribosomal RNA was depleted using the GenSeq rRNA Removal Kit (GenSeq, Shanghai, China). Following rRNA removal, the RNA was treated with bisulfite and purified using the EZ RNA Methylation Kit (Zymo Research, USA, Cat. No. R5001). Sequencing libraries were then constructed with the GenSeq Low‐Input RNA Library Prep Kit according to the manufacturer's instructions (GenSeq, Shanghai, China). Library quality was evaluated by assessing concentration and size distribution on the BioAnalyzer 2100 system (Agilent Technologies, USA). All libraries were sequenced on an Illumina NovaSeq 6000 platform with a paired‐end 150 bp configuration. Adapter and primer sequences were trimmed from raw reads using cutadapt (v5.0). Clean reads were aligned to the hg38 reference genome using meRanGs under default parameters. The m^5^C methylation sites were identified with meRanCall, and differentially methylated sites (DMSs) were detected using meRanCompare. Finally, methylation sites and DMSs overlapping mRNA exons were extracted using in‐house scripts.

### 5.17 RNA Sequencing (RNA‐seq)

In brief, total RNA was extracted from RA FLSs using the Trizol method (ThermoFisher, USA, Cat. No. 15596026CN). mRNA was purified with the GenSeq mRNA Purification Kit (GenSeq, Shanghai, China) according to the manufacturer's instructions. Library preparation was performed using the GenSeq Directional RNA Library Prep Kit as per the manufacturer's protocol. Library quality was evaluated by assessing concentration and size distribution on the BioAnalyzer 2100 system (Agilent Technologies, USA). All libraries were sequenced on an Illumina NovaSeq 6000 system in paired‐end 2 × 150 bp mode. For data analysis, adapter and primer sequences were trimmed from raw reads using cutadapt (v5.0). Clean reads were aligned to the hg38 reference genome using HISAT2 (v2.2.1) under default parameters. Read counts were generated with HTSeq, and gene expression levels were normalized using FPKM (Fragments Per Kilobase Million) to enable cross‐sample mRNA expression comparison. Differential expression analysis was conducted with the R package DESeq2. Functional enrichment analyses, including Gene Ontology and KEGG pathway analyses, were performed using the R package clusterProfiler (v4.16.0).

### 5.18 RNA Stability

For assessing mRNA stability in NSUN2‐knockdown and control RA FLSs, actinomycin D (Selleck, USA, S8964) was added at a concentration of 5 µg/mL. RNA was harvested at the indicated time points and subjected to RT‐qPCR analysis.

### 5.19 m^5^C‐MeRIP‐qPCR

Total cellular RNA was isolated using the TRIzol reagent. mRNA was enriched from total RNA via oligo (dT) magnetic bead‐based purification (Bio‐linkedin, China, NK‐1001). For controlled fragmentation, 18 µL of mRNA (dissolved in nuclease‐free water) was mixed with 2 µL of 10×RNA fragmentation buffer and incubated at 90°C for 30 s. The reaction was immediately terminated by adding 2 µL of fragmentation stop buffer, followed by chilling on ice. Fragmented mRNA was purified using the Zymo RNA Clean & Concentrator‐25 kit (ZYMO Research, USA, R1015) according to the manufacturer's instructions. m^5^C‐modified mRNA fragments were immunoprecipitated using an anti‐m^5^C antibody‐conjugated magnetic bead approach, following the established MeRIP protocol [41]. Briefly, fragmented mRNA was incubated with anti‐m^5^C antibody‐conjugated magnetic beads at 4°C for 4 h. Immunoprecipitated RNA was recovered using TRIzol reagent. Enrichment of m^5^C‐methylated transcripts was validated by RT‐qPCR using SYBR Green Master Mix and gene‐specific primers.

### 5.20 Luciferase Reporter Assay

Luciferase activity was determined using Firefly & Renilla Luciferase Reporter Assay Kit (meilunbio, China, MA0518‐1) following the manufacturer's protocol. The control vector, wild ICMT (ICMT‐WT), mutant ICMT (ICMT‐MUT) plasmid was synthesized by Genechem (Shanghai, China). The primer sequences of plasmid construction are listed in Table S6. ICMT‐WT or ICMT‐MUT plasmids were transfected into HEK‐293Ts infected with NSUN2 OE or control lentiviruses. Luciferase activities were detected 48 h after transfection by Varioskan LUX (Thermo Scientific, USA).

### 5.21 High‐Throughput Virtual Screening

To identify potential inhibitors targeting the CYS321‐containing pocket in human NSUN2, molecular docking simulations were performed using Schrödinger Maestro 12.8, with PyMOL employed for visualization and 3D mapping. The predicted NSUN2 structure was retrieved from the AlphaFold database (AlphaFold ID: AF‐Q08J23‐F1, https://alphafold.ebi.ac.uk/). The protein was hydrogenated and optimized (OPLS2005 force field, RMSD = 0.3 Å) by using the Protein Preparation Wizard module. A grid file was generated from the processed NSUN2 protein using the Receptor Grid Generation tool, centered on the CYS321‐containing pocket with dimensions of 20 Å × 20 Å × 20 Å. The 2D structures of compounds from the MCE Bioactive Compound Library Plus (19,100 compounds) were processed via the LigPrep module for hydrogenation, energy minimization, and 3D structure generation. Final molecular docking between the receptor and ligands was performed using the Glide‐based Virtual Screening Workflow module to identify candidate compounds. Following three‐tier molecular docking (high‐throughput virtual screening [HTVS], standard precision [SP], and extra precision [XP] modes), the top 50 compounds targeting NSUN2 were selected. To refine compound ranking, the binding affinity of each compound to NSUN2 was manually evaluated.

### 5.22 Surface Plasmon Resonance (SPR) Assays

SPR experiments were performed on a Biacore T200(GE Healthcare, USA) device at 25°C, with running buffer consisting of 1*PBS‐P+ (Cytiva, USA, BR‐28‐9950‐84, pH 7.4) supplemented with 5% DMSO. Briefly, NSUN2 protein was diluted to 50 µg/mL in 10 mM sodium acetate (pH 4.5) and immobilized onto channel 2 of a CM5 sensor chip at a flow rate of 10 µL/min. For kinetic measurements, each candidate compound (100 µM or varying concentrations) was injected over the chip surface for 150 s at a flow rate of 10 µL/min, followed by a 5‐min dissociation phase. Binding kinetics were recorded and analyzed using Biacore Insight Evaluation software with a 1:1 steady‐state affinity model.

### 5.23 Preparation and Characterizations of Cerium–Ce/SAA NPs

Ce/SAA NPs were synthesized via a self‐assembly method. Briefly, an aqueous solution of 1 mM SAA was mixed with 3 mM (NH_4_)_2_Ce(NO_3_)_6_ in methanol under stirring. The reaction proceeded for 2 h at room temperature. The resulting Ce/SAA NPs were collected by centrifugation and washed three times with methanol, followed by deionized water.

For characterization of Ce/SAA NPs, a transmission electron microscope (TEM, Japan, JEM‐2100) was used for imaging at an accelerating voltage of 200 kV. UV–vis spectra were acquired with a Shimadzu UV‐3600 spectrophotometer. Hydrodynamic size and zeta potential were measured using a Malvern Zetasizer Nano ZSP (Malvern Instruments, UK). Fourier‐transform infrared (FT‐IR) spectra were recorded on a PerkinElmer Spectrum 100 spectrometer (PerkinElmer). X‐ray photoelectron spectroscopy (XPS) measurements were performed on an Axis Ultra DLD multifunctional photoelectron spectrometer (Kratos, UK). The cerium content was quantified by inductively coupled plasma–mass spectrometry (ICP‐MS, Agilent 8900, USA). Electron spin resonance (ESR) spectra were collected on a Bruker EMXplus ESR spectrometer (Germany).

### 5.24 Degradation of Ce/SAA NPs

Ce/SAA NPs (0.2 mg) were dispersed in 5 mL of PBS at 37°C, adjusted to either pH 5.2 (simulating the arthritic inflammatory environment) or pH 7.4 (mimicking the human physiological microenvironment), and stirred for 6, 12, 24, 36, or 48 h. At each time point, the dispersions were centrifuged to separate released Ce ions or SAA from the nanoparticles. The Ce ions concentration in the supernatant was quantified by ICP‐MS, and the SAA concentration was determined by UV‐vis spectrophotometry at 288 nm.

### 5.25 In Vitro Characterization of ROS Scavenging Capability

Hydroxyl radicals (•OH) were generated via the Fenton reaction in a 50 µL aqueous solution containing 10 µL of 9 mM H_2_O_2_, 10 µL of 2 mM FeSO_4_, 2 µL DMPO, and Ce/SAA NPs at a concentration of 50 µg/mL. Electron spin resonance (ESR) spectra were recorded for the mixtures H_2_O_2_ + Fe^2^^+^ and H_2_O_2_ + Fe^2^^+^ + Ce‐SA NPs (50 µg/mL), using 5,5‐dimethyl‐1‐pyrroline‐N‐oxide (DMPO) as a spin‐trapping agent for •OH.

### 5.26 Preparation of Ce/SAA NPs or ^RhB^Ce/SAA NPs

Ce/SAA NPs were synthesized via a self‐assembly method. Briefly, an aqueous solution of 1 mM SAA was mixed with 3 mM (NH_4_)_2_Ce(NO_3_)_6_ in methanol under stirring. The reaction proceeded for 2 h at room temperature. The resulting Ce/SAA NPs were collected by centrifugation and washed three times with methanol followed by deionized water. To prepare ^RhB^Ce/SAA NPs, 20 mg of Ce/SAA NPs dispersion was incubated with 1 mg of DSPE‐PEG‐RhB for 12 h. The mixture was then purified by centrifugation and washed three times with deionized water to yield the final ^RhB^Ce/SAA NPs.

### 5.27 Therapeutic Intra‐Articular NSUN2 Knockdown in Collagen‐Induced Arthritic Rats

To establish the CIA model in rats, male Wistar rats received an intradermal injection of bovine type II collagen (2 mg/mL; Chondrex, USA, 20022) emulsified 1:1 (vol/vol) with complete Freund's adjuvant (5 mg/mL; Chondrex, USA, 7023) at the tail base. One week later, a booster injection was administered intradermally using the same collagen/adjuvant emulsion. For local NSUN2 knockdown in joints, NSUN2 siRNA (siNsun2) or control siRNA (NC; RiboBio, Guangzhou, China) was injected intra‐articularly on day 8 and 15 postinitial immunization. Arthritis progression was monitored at 3‐day intervals.

### 5.28 SAA Treatment in CIA Mice

Briefly, CIA was induced in 8‐week‐old male DBA/1J mice via intradermal immunization at the base of the tail with 200 µg bovine type II collagen emulsified at a 1:1 volume ratio in complete Freund's adjuvant. On day 21, the mice received an intraperitoneal booster injection of bovine type II collagen. Starting from day 22, the mice were randomly assigned to receive intraperitoneal injections every 2 days of either free SAA (20 mg/kg or 40 mg/kg), Ce/SAA NPs (20 mg/kg or 40 mg/kg), ammonium cerium nitrate (Ce), or vehicle control (DMSO). Arthritis severity and progression were monitored every 3 days using arthritis scores before and during treatment. All mice were anesthetized on day 37, and hind paws were dissected and fixed in 4% paraformaldehyde for histological analysis.

### 5.29 Biodistribution Analysis

To determine the in vivo biodistribution of Ce/SAA nanoparticles, CIA mice (*n* = 3) were administered the nanoparticles intravenously at an SAA concentration of 40 mg/kg. At 24 h postinjection, mice were euthanized, and joint tissues together with major organs (heart, liver, spleen, lung, and kidney) were excised and detected for cerium element contents via inductively coupled plasma‐mass spectrometry (ICP‐MS).

### 5.30 Micro‐CT Scan

Rats were euthanized by an intraperitoneal injection of 150 mg/kg pentobarbital sodium. Hind paws and ankle joints were carefully harvested from CIA rats and scanned on day 23 using a SkyScan 1276 micro‐CT system (Bruker, Germany) at 70 kV/200 µA with a 18 µm voxel size and 0.5 mm aluminum filter. The 3D reconstruction with quantitative analysis was generated using the manufacturer‐supplied 3‐matic software (Bruker, Germany). The region of interest (ROI) within the ankle bone was manually delineated. The bone volume/tissue volume (BV/TV), bone surface to bone volume ratio (BS/BV), trabecular thickness (Tb.Th), trabecular separation (Tb.Sp), and trabecular number (Tb.N) were measured using CT Analyser program (Bruker, Germany).

### 5.31 Histological Changes

Following treatment, rats and mice were euthanized. Rat joints or mouse paws were fixed in 4% formalin for 48 h, then decalcified in 10% EDTA‐2Na. The decalcified samples were subsequently embedded in paraffin and sectioned into 5‐µm‐thick slices. Tissue sections were stained with hematoxylin and eosin (H&E) and safranin O using standard protocols. Histopathological features, including extra‐articular infiltration, synovitis, cartilage destruction, and bone erosion, were scored as separate parameters by two observers in a blinded manner. Pathological changes in the liver and kidney were also evaluated via H&E staining. All animal experiments were approved by the Animal Experimental Ethics Committee of the First Affiliated Hospital of Sun Yat‐sen University.

### 5.32 Immunohistochemical Staining

Paraffin‐embedded tissue sections were deparaffinized, rehydrated, and subjected to heat‐mediated antigen retrieval in citric acid buffer. Slides were then treated with 3% H_2_O_2_ for 10 min to quench endogenous peroxidase activity, followed by 1 h blocking with 5% BSA in PBS. After overnight incubation with primary antibodies (NSUN2 (Abcam, UK, ab259941), ICMT (Solarbio, China, K001926P)) at 4°C, sections were stained with species‐matched HRP‐conjugated secondary antibodies. Immunoreactivity was developed using diaminobenzidine (DAB) (Beyotime, China, P0203) substrate, counterstained with hematoxylin, and imaged using an Olympus BX63 microscope (Japan).

### 5.33 Prediction of Potential RBP‐Binding Motifs

Potential RBP‐binding motifs within ICMT mRNA were scanned using FIMO with a *p*‐value cutoff of 0.001 to prioritize candidate RBPs for subsequent validation [23].

### 5.34 Statistical Analyses

Data are derived from at least 3 independent experiments and are presented as the mean ± SD for the in vitro experiments. The quantitative data of immunoblots and mRNA expression relative to GAPDH, β‐actin, or β‐tubulin levels, normalized to expression in the control. Data were analyzed by 2‐tailed Student's *t*‐test between two‐group comparisons; three or more different groups were evaluated by one‐way ANOVA followed by Bonferroni's post hoc comparisons. All statistical analyses were performed using GraphPad Prism 8.0 (USA, RRID:SCR_002798) and a *p‐*value less than 0.05 was considered significant. The experimental procedures and data analyses were performed in a blinded manner.

### 5.35 Declaration of Generative AI and AI‐Assisted Technologies

During the preparation of this work, the author(s) used AI for language editing and grammar correction of selected portions of the manuscript. The author(s) reviewed and revised all AI‐assisted content. The author(s) take full responsibility for the content of the published article.

## Author Contributions

Ruiru Li, Simin Chen, Huijuan Hu, and Shiyao Wu performed the investigation (the majority of the experiments) and formal analysis (data analysis and interpretation). Kai Sun, Chenxi Peng, and Suling Liu performed an investigation (in vivo experiments). Wang Jingnan, Qian Qiu, Yu Kuang, Liuqin Liang, and Shuoyang Zhang provided resources (clinical sample collection) and data curation. Yu Wen and Wei Chen contributed to conceptualization (study concept and design). Hanshi Xu and Youjun Xiao contributed to conceptualization, supervision, funding acquisition, and writing (original draft, review & editing). All authors have reviewed and approved the final manuscript.

## Funding

This work was supported by the National Natural Science Foundation of China (grant numbers 82271818, 82471824 to Hanshi Xu, 82071831, 82572051 to Youjun Xiao, 82302022 to Ruiru Li, 82502161 to Yu Kuang, China); and the Guangdong Basic and Applied Basic Research Foundation (grant numbers 2024A1515012944 to Youjun Xiao, 2023A1515011768 to Hanshi Xu, China).

## Conflicts of Interest

The authors declare no conflicts of interest.

## Supporting information




**Supporting File**: advs76401‐sup‐0001‐SuppMat.docx.

## Data Availability

The raw sequence data (including RNA‐seq and m^5^C BS‐seq) reported in this paper have been deposited in the Genome Sequence Archive (Genomics, Proteomics & Bioinformatics 2025) in National Genomics Data Center (Nucleic Acids Res 2025), China National Center for Bioinformation / Beijing Institute of Genomics, Chinese Academy of Sciences under accession number GSA‐Human: HRA014380. The data will be publicly available at https://ngdc.cncb.ac.cn/gsa‐human on October 29, 2027.
