# Supplementary material for: Cerium Nanoparticle‐Mediated Inhibition of the NSUN2/m5C Axis Suppresses Synovial Aggression in Rheumatoid Arthritis
Source: Adv Sci (Weinh). 2026 Jul 3;13(54):e76401. doi: 10.1002/advs.76401 (PMC13334591; doi:10.1002/advs.76401)
Supplement: Supplementary file 1 [file ADVS-13-e76401-s001.docx]

**Supplemental Figure**

**Fig. S1.** **NSUN2 expression in CIA model and effect of treatment with IL-1β, TNF-α or DXM on NSUN2.**

(A) Immunohistochemistry was used to detect NSUN2 protein expression in synovium from mice (original magnification, ×100).

(B) Effect of treatment with TNF-α, IL-1β, DXM or MTX on NSUN2. RA FLSs were treated with TNF-α (10 ng/mL), IL-1β (10 ng/mL) for the indicated times,or with DXM (0.1, 1, 10 μg/mL) or MTX (5, 10, 20 μg/mL) for 24 h. Western blot were used to analyze NSUN2 protein expression.

Data are presented as the means ± SD from at least 4 independent experiments. ^*^*P* < 0.05, ^**^*P* < 0.01, ^***^*P* < 0.001 versus 0h or CTR by one-way ANOVA with Bonferroni’s post-hoc comparison. CIA: collagen-induced arthritis; TNF-α: tumor necrosis factor-α; IL-1β: interleukin-1β; DXM: dexamethasone; MTX: methotrexate; CTR: untreated control cells.

**Fig. S2. Validation of NSUN2 knockdown/overexpression efficiency and functional consequences of NSUN2 on RA FLSs proliferation, apoptosis, proinflammatory cytokine expression, and MMPs expression.**

(A) Efficiency of NSUN2 knockdown by siRNA in RA FLSs. Cells were transfected with control siRNA or NSUN2 siRNA for 48 h; silencing efficiency was determined by RT-qPCR and Western blot.

(B) The secretion of MMPs was detected by ELISA.

(C) EdU staining (left) and quantitative analysis (right) of proliferating RA FLSs (original magnification, ×100).

(D) Cell apoptosis analyzed by Annexin V/PI staining via flow cytometry (left) with quantitative analysis (right).

(E) mRNA expression of IL-1β, IL-6, and IL-8 in RA FLSs treated with TNF-α (10 ng/mL) for 24 h, detected by RT-qPCR.

(F) Infection efficiency of lentiviruses encoding NSUN2 variants in RA FLSs. Cells were transduced with empty vector control (Vector), wild-type NSUN2 overexpression (NSUN2-OE), or NSUN2 double mutant (NSUN2-DM) lentiviruses for 96 h.

(G) Schematic representation of targeted mutagenesis in NSUN2, showing substitution of cysteine residues at positions 271 and 321 with alanine (C271A/C321A).

Data are presented as the means ± SD from at least 4 independent experiments. ^*^*P*<0.05, ^**^*P*<0.01 and ^***^*P*<0.001 versus siC or Vector; by one-way ANOVA with Bonferroni’s post-hoc comparison. MMP: matrix metalloproteinase; TNFα: tumor necrosis factor-α; OE: overexpression; DM: double mutant.

**Fig. S3. Effects of reader-related RBPs on ICMT mRNA expression in RA FLSs.**

(A-D) RT-qPCR was used to confirm the knockdown efficiency of YBX1, ALYREF, SRSF2, and SRSF10 and to analyze ICMT mRNA expression after knockdown of the indicated RBPs. Data are presented as the mean ± SD from at least 5 independent experiments. ****P*< 0.001 versus siC indicates knockdown efficiency of the indicated RBPs, ^###^*P*< 0.001 versus siC indicates increased ICMT mRNA expression after ALYREF knockdown, as determined by one-way ANOVA followed by Bonferroni’s post hoc test. siC, control siRNA; RBP, RNA-binding protein.

**Fig. S4. Effect of TNF‑α and IL‑1β on ICMT expression, and validation of ICMT knockdown/overexpression efficiency and functional consequences of ICMT overexpression on RA FLSs migration, invasion and MMPs expression.**

(A) Effect of treatment with TNF-α and IL-1β on ICMT. RA FLSs were treated with TNF-α (10 ng/mL) or IL-1β (10 ng/mL). Western blot were used to analyze ICMT protein expression.

(B) Efficiency of siRNA-induced ICMT knockdown. RA FLSs were transfected with control siRNA (siC) or ICMT siRNA (siICMT) for 48 h, and silencing efficiency was measured by RT-qPCR and Western blot (with the quantification presented in the right panel).

(C) Infection efficiency of ICMT overexpression lentivirus in RA FLSs. RA FLSs infected with control lentivirus (Vector) or ICMT overexpression lentivirus (ICMT OE) for 96 h were analyzed for ICMT mRNA and protein expression by RT-qPCR and Western blot (with the quantification presented in the right panel), respectively.

(D-E) Effect of ICMT overexpression on cell migration, and invasion in RA FLSs. The migration and invasion ability of RA FLSs was analyzed by Transwell assays (original magnification, ×100) (D) and wound healing of RA FLSs (original magnification, ×50) (E).

(F) RT-qPCR was used to detect MMP1, MMP3, and MMP9 mRNA expression.

(G) Knockdown efficiency of NSUN2 shRNA lentivirus. RA FLSs infected with scramble shRNA lentivirus (Ctrl) or NSUN2-targeting shRNA lentivirus (shNSUN2) for 96 h were analyzed for NSUN2 mRNA and protein expression by RT-qPCR assays and Western blot (quantification shown in right panel), respectively.

Data are presented as the means ± SD from at least 3 independent experiments. ^*^*P*<0.05, ^**^*P*<0.01 and ^***^*P*<0.001 versus 0h, siC, Vector or Ctrl, by Student’s t-test or one-way ANOVA with Bonferroni’s post-hoc comparison. TNF-α: tumor necrosis factor-α; IL-1β: interleukin-1β; CTR: control; MMP: matrix metalloproteinase; OE: overexpression.

**Fig.** **S5. Effect of SAA on NSUN2 expression in RA FLSs.**

Effect of SAA on NSUN2 mRNA (A) and protein (B) expression in RA FLSs, analyzed by RT-qPCR and Western blot, respectively. Data are presented as the means ± SD from at least 3 independent experiments. No significant difference versus DMSO, by one-way ANOVA with Bonferroni’s post-hoc comparison.

**Fig. S6.** **Characterization of Ce/SAA NPs.**

(A) Synthesis of Ce/SAA NPs via one-step method of SAA and Ce^4+^.

(B-C) TEM Image (B), and hydrodynamic size distribution (C) of Ce/SAA NPs.

(D-E) Hydrodynamic size change distribution of Ce/SAA NPs in PBS (D) and DMEM (E).

(F) Thermogravity curves of Ce/SAA NPs.

(G) Biodistribution of Ce/SAA NPs 24 h after intravenous injection in CIA mice as determined by ICP-MS.

Data are presented as the means ± SD from 3 independent experiments. SAA: salvianolic acid A; NPs: nanoparticles; TEM: transmission electron microscope; ICP-MS: inductively coupled plasma-mass spectrometry

**Fig. S7.** **In vivo safety and biocompatibility evaluation of SAA and Ce/SAA NPs.**

(A) H&E staining was used to show the pathological changes of the liver and kidney in control mice or CIA mice treated with DMSO, Ce alone, free SAA (20 or 40 mg/kg), or Ce/SAA NPs (20 or 40 mg/kg).

(B) Serum levels of alanine aminotransferase (ALT), aspartate aminotransferase (AST), and creatinine (Scr) in CIA mice treated with DMSO, Ce alone, free SAA (20 or 40 mg/kg), or Ce/SAA NPs (20 or 40 mg/kg) (n = 5 per group).

Data represent means ± SD from 5 independent experiments, by one-way ANOVA with Bonferroni’s post-hoc comparison. ALT: alanine aminotransferase; AST: aspartate aminotransferase; Scr: creatinine; CIA: collagen-induced arthritis

**Supplemental Table**

| Demographic and clinical features | Values |
| --- | --- |
| Age, yrs (mean±SD) | 56.88±10.505 |
| Female, n(%) | 21(84) |
| Male, n(%) | 4(16) |
| Disease duration, yrs (mean±SD) | 9.8840±10.98498 |
| Rheumatoid factor-positive, n(%) | 19(76) |
| Anti-CCP-positive, n(%) | 19(76) |
| DAS28(ESR) (mean±SD） | 5.3492±1.27716 |
| Previous medications, n(%) |  |
| Prednisone(<10mg/d) | 12(48) |
| Methotrexate | 5(20) |
| Leflunomide | 2(8) |
| Sulfasalazine | 0(0) |
| Hydroxychloroquine | 4(16) |

Table S1. Demographic and clinical features of patients with active RA

| Demographic and clinical features | Values |
| --- | --- |
| Age, yrs (mean±SD) | 49±13.56466 |
| Female, n(%) | 9(64.29) |
| Male, n(%) | 5(35.71) |

Table S2. Demographic and clinical features of HC subjects

| shRNA | Number | Sequences |
| --- | --- | --- |
| h-NSUN2 | 1 | CCCAAGAATGAACGGCTTCAT |
|  | 2 | GAGCGATGCCTTAGGATATTA |
|  | 3 | CAGTGGAAGGTAATGACGAAA |

Table S3. Sequences of shRNA

| siRNA | Number | Sequences |
| --- | --- | --- |
| h-NSUN2 | 1 | GGGTTATCCTCACAAATGA |
|  | 2 | GCATCATGGTGGTCAACCA |
| h-ICMT | 1 | GAAGAAGAAATCTCACTAA |
|  | 2 | CTTCCGCGATCGAACAGAA |
| h-YBX1 | 1 | GGAACGGATATGGTTTCAT |
|  | 2 | CAAGGAAGATGTATTTGTA |
| h-ALYREF | 1 | GGAAACUGCUGGUGUCCAA |
|  | 2 | CUCUGGACGACAUCAUUAA |
| rat-Nsun2 | 1 | GGAAGAAATGGACTACCTT |
|  | 2 | CTGAAGTACGAACCAGATT |
|  | 3 | GAAGATGAAGGTCATTAAC |

Table S4. Sequences of siRNA

| Name | Forward | Reverse |
| --- | --- | --- |
| h-β-actin | TCAAGATCATTGCTCCTCCTGAG | ACATCTGCTGGAAGGTGGACA |
| h-NSUN2 | TGGCTGAAGGTGGAAGGATG | ATCTGGGTGTGTCTGCTGTG |
| h-ICMT(MeRIP-qPCR) | GAAGTGCCTACTCTCTGGGC | CAGATGGCTGGGTCTTCCAA |
| h-NSUN6 | ATCTGCGTCCGTTTCACCAC | GGCTTCCACCACACCTCATC |
| h-TET1 | CAGGACCAAGTGTTGCTGCTGT | GACACCCATGAGAGCTTTTCCC |
| h-TET2 | GCTTACCGAGACGCTGAGGAAA | AGAGAAGGAGGCACCACAGGTT |
| h-TET3 | CCACAAGGACCAGCATAACCTC | CTCGCTACCAAACTCATCCGTG |
| h-MMP1 | CTCTGGAGTAATGTCACACCTCT | TGTTGGTCCACCTTTCATCTTC |
| h-MMP3 | AAAGACAGGCACTTTTGGCG | CTTCATATGCGGCATCCACG |
| h-MMP9 | TGTACCGCTATGGTTACACTCG | GGCAGGGACAGTTGCTTCT |
| h-IL-1β | ATGATGGCTTATTACAGTGGCAA | GTCGGAGATTCGTAGCTGGA |
| h-IL-6 | ACTCACCTCTTCAGAACGAATTG | CCATCTTTGGAAGGTTCAGGTTG |
| h-IL-8 | ACTGAGAGTGATTGAGAGTGGAC | AACCCTCTGCACCCAGTTTTC |
| h-ICMT | CGCTTGGTTTCGGCATCCTTCT | CGGAAGAATCGCCACACTGTCA |
| h-CALM1 | CCAACAGAAGCTGAATTGCAGGA | CAAAGACTCGGAATGCCTCACG |
| h-MOXD1 | GCACTTTGGAGTGCCTGGAAGA | AATGACGCAGCCTGATGCCTCT |
| h-ANPEP | GCTGTTTGACGCCATCTCCTAC | GTTCTGGTAGGCAAAGGTGTGG |
| h-MRPS6 | CGCTTCCTTATAGGATCTCTGCC | GAGACAAGTGCTCCACCATGCT |
| rat-Nsun2 | AGCAGTTTTGGGGACCGTTT | GGGCTTGGTTCTCTCTCTCC |
| rat-GAPDH | AGTGCCAGCCTCGTCTCATA | GATGGTGATGGGTTTCCCGT |
| h-YBX1 | CTTCATTGCCGTCCTCTCTAGG | GCAGGAGAACAAGGTAGACCAG |
| h-ALYREF | GGAGTCTCAGACGCCGATATTC | GCATCTGCCTTCCGCTCAAAGT |

Table S5. The primer sequences of mRNA

| plasmid | Sequences |
| --- | --- |
| **GV272-REPORT-ICMT-CDS-WT (wild-type CDS of ICMT)** | CTTTTGTAGTGGTTTGGCAAGGTTAGAAGGCCTCGGCCTCTCTGTCATGCTGGGAAGTGCCTACTCTCTGGGCCACTGCTGCAGAGGCCGTGGCACTTGT**C**ATGGGTTTGGAAGACCCAGCCATCTGCAGCAGAGGCAGCCTATCCCATTGCAAGGAGAGGAACTGAACGGAGTAATTATTCTACTCTTCTTTTTACATAA |
| **GV272-REPORT-ICMT-CDS-Mut (mutant CDS of ICMT, m^5^C was replaced by T in the m^5^C motifs)** | CTTTTGTAGTGGTTTGGCAAGGTTAGAAGGCCTCGGCCTCTCTGTCATGCTGGGAAGTGCCTACTCTCTGGGCCACTGCTGCAGAGGCCGTGGCACTTGT**T**ATGGGTTTGGAAGACCCAGCCATCTGCAGCAGAGGCAGCCTATCCCATTGCAAGGAGAGGAACTGAACGGAGTAATTATTCTACTCTTCTTTTTACATAA |

Table S6. Sequences of ICMT-CDS of plasmid used in luciferase reporter assay
